# Bioactive sorbicillinoid derivatives from an endophytic fungus *Trichoderma citrinoviride*

**DOI:** 10.3389/fmicb.2025.1485032

**Published:** 2025-01-28

**Authors:** Yan-Ping Xia, Yan Xie, Li Rao, Guo-Ping Yin

**Affiliations:** ^1^Department of Pharmacy, Nanan People’s Hospital of Chongqing, Chongqing, China; ^2^Department of Pharmacy, Zhengzhou Shuqing Medical College, Zhengzhou, China; ^3^Chongqing Key Laboratory of High Active Traditional Chinese Drug Delivery System, Chongqing Medical and Pharmaceutical College, Chongqing, China; ^4^Engineering Research Center of Coptis Development and Utilization (Ministry of Education), College of Pharmaceutical Sciences and Chinese Medicine, Southwest University, Chongqing, China

**Keywords:** sorbicillinoid, fungus, Trichoderma, antioxidant, anti-inflammatory

## Abstract

Three new sorbicillinoid derivatives, citrinsorbicillinol A-C (**1**–**3**), along with three known compounds, such as trichosorbicillin G (**4**), dibutyl phthalate (**5**), and 3-(4-methoxyphenyl) propanoic acid (**6**), were isolated from the endophyte *Trichoderma citrinoviride* of *Coptis chinensis*. Their structures were elucidated through extensive analyses of spectroscopic data, computer-assisted structure elucidation (ACD/Structure Elucidator), density functional theory (DFT) calculations of the nuclear magnetic resonance (NMR) spectra, and electronic circular dichroism (ECD). Biologically, compounds **1**–**4** exhibited potential antioxidant activity, as assessed using the 1,1-diphenyl-2-picrylhydrazyl (DPPH) assay, with IC_50_ values ranging from 27.8 to 89.6 μM. In particular, compounds **2** and **3** demonstrated radical scavenging activity comparable to that of the positive control, ascorbic acid, with IC_50_ values of 27.8 and 31.2 μM, respectively. Moreover, compound **1** exhibited potential anti-inflammatory activity by inhibiting nitric oxide (NO) production in lipopolysaccharide (LPS)-induced RAW 264.7 macrophages, with an IC_50_ value of 52.7 μM. These findings underscore the therapeutic potential of the new sorbicillinoid derivatives for antioxidant and anti-inflammatory applications.

## 1 Introduction

Fungal secondary metabolites are crucial due to their broad chemical diversity and biological activities, making them highly valuable in fields such as medicine (Cai et al., 2024). In particular, fungi from extreme environments, such as endophytes in medicinal plants and marine fungi, often have the ability to produce novel secondary metabolites (Barzkar et al., 2024; Yu et al., 2024). It is essential to thoroughly explore these rare fungi, as this could lead to the discovery of new compounds with unprecedented properties, thereby fully unlocking their potential and advancing our understanding and utilization of fungal secondary metabolites. *Coptis chinensis* Franch., known for its dried rhizomes as a source of the traditional Chinese medicine “Huang lian,” primarily grows in high-altitude areas (1,500–1,800 meters) such as Chongqing, Sichuan, and Hubei provinces in China. Pharmacological studies have shown that its chemical constituents, mainly alkaloids such as berberine, possess significant biological activities, including antibacterial, antitumor, and antidiabetic effects. The high-altitude environment, along with its internal chemical conditions, provides a unique habitat for endophytic fungi, which may produce distinctive secondary metabolites. However, there have been few reports on the secondary metabolites of endophytic fungi in *Coptis chinensis* to date (Zhang et al., 2018; Yin et al., 2021a; Yin et al., 2024).

Sorbicillinoids, a family of metabolites with hexaketide structures, are primarily derived from fungal sources (Xie et al., 2021; Peng et al., 2022). These structures feature a flexible hexacyclic ring and a sorbyl side chain that can undergo reactions such as the Michael addition or the Diels–Alder reaction, resulting in highly oxygenated and polycyclic carbon skeletons (Kahlert et al., 2020; Sib and Gulder, 2017; Rehman et al., 2020). To date, nearly 195 naturally occurring sorbicillinoids have been identified. They are recognized for their diverse biological activities, including anticancer effects, radical scavenging properties, and antibacterial activity (Chen et al., 2022; Wang et al., 2022; Zhao et al., 2022; Wang et al., 2023; Yin et al., 2024; Ying et al., 2024; Zhang et al., 2024a; Zhang et al., 2024b). In our ongoing endeavor to search for structurally unique and biologically interesting metabolites from fungal resources (Yin et al., 2021a; Yin et al., 2024; Wang et al., 2024), the fungus *Trichoderma citrinoviride* was isolated from the rhizomes of a 5-year-old *Coptis chinensis* plant collected from Shizhu, Chongqing. Through solid-state fermentation using rice, six compounds were isolated and identified, including three new sorbicillinoid derivatives, citrinsorbicillinol A-C (**1**–**3**), along with three known compounds—trichosorbicillin G (**4**), dibutyl phthalate (**5**), and 3-(4-Methoxyphenyl) propanoic acid (**6**). Herein, the details of their isolation, structural elucidation, and bioactivities are presented.

## 2 Materials and methods

### 2.1 General experimental procedures

UV spectra were recorded using a UV-2450 visible spectrophotometer (Shimadzu, Japan). IR spectra (KBr disks) were obtained using a Shimadzu IRPrestige-21 instrument (Shimadzu, Japan). A JASCO J-815 spectropolarimeter was used to measure electronic circular dichroism (ECD) spectra (JASCO, Japan). Nuclear magnetic resonance (NMR) spectra were recorded on a Bruker Advance III 400 spectrometer (Bruker, Germany), with tetramethylsilane (TMS) as the internal standard. High-resolution electrospray ionization mass spectra (HRESIMS) were acquired using a Bruker impact II Q-TOF mass spectrometer (Bruker, Germany) and an Agilent 6520B mass spectrometer (Agilent, American). Analytical high-performance liquid chromatography (HPLC) was conducted with a Shimadzu LC-20 AD pump and a SPD-M20A UV detector (Shimadzu, Japan), using a YMC RP-C18 column (5 μm, 4.6 × 250 mm). Preparative high-performance liquid chromatography (HPLC) was performed on a Separation LC-UV system (Separation, China) using a YMC RP-C18 column (5 μm, 10 × 250 mm). The flow rate was set at 3.0 ml/min, and detection was carried out at wavelengths of 210 nm and 254 nm using a dual-channel UV detector. Column chromatography was performed using silica gel (100–200 and 200–300 mesh, Qingdao Marine Chemical Inc., China), MCI (50 μm, Mitsubishi, Japan), and Sephadex LH-20 (Pharmacia Fine Chemical Co., Ltd.).

### 2.2 Fungal material and identification

The endophytic fungus was obtained from the traditional Chinese medicinal herb *Coptis chinensis* Franch. through the plate coating method, which were collected from Shizhu, Chongqing, China. The isolated strain was identified as *Trichoderma citrinoviride* based on morphological characteristics, and this identification was further supported by 18S rDNA and internal transcribed spacer (ITS) sequences, which showed 100% identity to the known *Trichoderma citrinoviride* (GenBank Accession KY750459.1). The basic characteristics of *Trichoderma citrinoviride* growth are as follows (Supplementary Figures S29, S30): When *Trichoderma citrinoviride* is inoculated onto a potato dextrose agar (PDA) medium at 28°C, the colony grows rapidly and exhibits aerial mycelium. During the first 1–3 days, the colony appears light green on the surface and yellowish-green on the reverse side, with a transparent mycelium. By the 4th day, extensive areas of white, fluffy colonies begin to appear. By the 5th day, the colonies become a cottony, olive-green mass. The mycelium is septate, and the conidiophores are characterized by a long main axis with shorter secondary branches, which are alternately arranged with unequal spacing and branching at acute or nearly right angles. Some of the terminal branches are flask-shaped, and the phialides bear smooth-walled spores. The conidia are colorless to green, ellipsoidal, and relatively small. The sequence information of this fungus is as follows:

GGATCACCTGATCCGAGGTCACATTTCAGAGTTTGGGGTGTTTTACGGCTGTGGCCGCGCCGCGCTCCCGGTGCGAGTGTGCAAACTACTGCGCAGGAGAGGCTGCGGCGAGACCGCCACTGTATTTCGGGGGCGGCCCGGTGAGGGGCCGATC CCCAACGCCGACCCCCCGGAGGGGTTCGAGGGTTGAAATGACG CTCGGACAGGCATGCCCGCCAGAATACTGGCGGGCGC AATGTGCGTTCAAAGATTCGATGATTCACTGAATTCTGCAATTCACATTACTTATCGCATTTCGCTGCGTTCTTCATCGATGCCAGAACCAAGAGATCCGTTGTTGAAAGTTTTGATTCATTTTCGAGACGCCCGCTAGGGTCGCCGAGAAAGGCTCAGAGCAAAAATAAAACAGAGCCGCGACGTAGGCCGCGACGGAGAGAAAAAAGAGTTTGAGTTGGTCCTCCGGCGGGCGCCATGGGATCCGGGGCTGCGACGCGCCCGGGGCAGAGAATCCCGCCGA GGCAACAGATTGGTAACGTTCACATTGGGTTTGGGAGTTGTAAACTCGGTAATGATCCCTCCGCTGGTTCACCAACGGAGACCTTGTT CCCTT.

### 2.3 Fermentation and extraction

The strain was cultured on potato dextrose agar (PDA) at 28°C for 7 days. Then, two pieces of the agar (about 1.0 cm^3^) were added to an Erlenmeyer flask (250 ml) containing 100 ml of potato dextrose liquid medium. The flask was then incubated on a rotary shaker at 28°C and 150 rpm for 5 days to prepare the seed culture. Solid fermentation was carried out in 400 Erlenmeyer flasks (1 L each). The flasks were sterilized by autoclaving prior to use and contained 200 g of rice, 1.0 g of glucose, 0.5 g of CuSO_4_•5H_2_0, and 200 ml of distilled water. All flasks were incubated at room temperature for 30 days. The solid cultures were extracted with ethyl acetate (EtOAc) three times at room temperature. The solvent was removed under reduced pressure to yield 1,000 g of crude extract.

### 2.4 Isolation of the metabolites

The crude extract (1,000 g) was subjected to silica gel column chromatography (CC), and elution was performed using a mixture of petroleum ether (boiling point, 60–90°C) and EtOAc in ratios ranging from 15:1 to 0:1, resulting in increasing polarity. This process yielded eight fractions (Fr.1–8), as determined by thin layer chromatography (TLC) analysis. Fr. 5 (150 g) was further fractionated by repeated CC on silica gel, again eluting with petroleum ether and EtOAc (15:1 to 0:1), to produce seven subfractions (Fr.5.1–5.7). Fr.5.3 (45 g) was subjected to additional silica gel CC, eluting with the same solvent system to generate five fractions (Fr.5.3.1–5.3.5). Fr.5.3.2 (9.7 g) was then separated using MCI CC with gradient elution (MeOH/H_2_O, 30:70 to 100:0), resulting in 154 subfractions (Fr.5.3.2.1–5.3.2.154). Specific subfractions were purified by semi-preparative HPLC as follows: Fr.5.3.2.12 (37.8 mg) was purified using ACN/H_2_O (38:62, 3 ml/min) to obtain compound **3** (12.1 mg, t_R_ = 19.3 min; proportion of total extract, 0.00121%). Fr.5.3.2.26 (25.6 mg) yielded compound **2** (12.3 mg, t_R_ = 55.2 min; proportion of total extract, 0.00123%) after purification with MeOH/H_2_O (63:37, 3 ml/min). Compound **6** (9.0 mg, t_R_ = 19.9 min; proportion of total extract, 0.0009%) was purified from Fr.5.3.2.28 (17.5 mg) using MeOH/H_2_O (43:57, 3 ml/min). Fr.5.3.2.48 (25.7 mg) resulted in compound **5** (11.9 mg, t_R_ = 22.2 min; proportion of total extract, 0.00119%) after purification with ACN/H_2_O (80:20, 3 ml/min). Separately, Fr.5.4 (7.6 g) was fractionated using Sephadex LH-20 with CH_2_Cl_2_-MeOH (1:1) to produce four subfractions (Fr.5.4.1–5.4.4). Fr.5.4.3 (3.1 g) was further separated by MCI CC through gradient elution with MeOH/H_2_O (30:70 to 100:0) to yield 21 subfractions (Fr.5.4.3.1–5.4.3.21). Compound **1** (15.3 mg, t_R_ = 67.4 min; proportion of total extract, 0.00153%) was purified from Fr.5.4.3.12 (35.2 mg) using MeOH/H_2_O (40:60, 3 ml/min). Finally, compound **4** (9.1 mg, t_R_ = 39.1 min; proportion of total extract, 0.00091%) was obtained from Fr.5.4.3.15 (31.2 mg) after purification with MeOH/H_2_O (50:50, 3 ml/min).

Citrinsorbicillinol A (**1**): yellow powder; UV (MeOH) (log *ε*) λ_max_ 229 (3.06), 261 (3.61), 282 (2.85), 366 (3.38) nm; IR (KBr) ν_max_/cm^−1^ 3,752, 3,689, 2,372, 1701, 1,655, 1,544, 1,386, 1,155, 1,107, 1,026; HRESIMS *m/z* 237.0799 [M + H]^+^ (calcd for C_12_H_13_O_5_^+^ 237.0763); ^1^H NMR and ^13^C NMR data, see Table 1.

**Table 1:** ^1^H NMR (400 MHz) and ^13^C NMR (100 MHz) spectroscopic data for compounds **1**–**3**.

Position	**1** (DMSO-*d*_6_)		Position	**2** (CDCl_3_)		**3** (CDCl_3_)	
	*δ*_H_ (*J* in Hz)	*δ*_C_		*δ*_H_ (*J* in Hz)	*δ*_C_	*δ*_H_ (*J* in Hz)	*δ*_C_
1		163.6	1		113.3		113.2
2		102.8	2	7.36 (s)	129.2	7.36 (s)	129.1
3		165.9	3		114.9		114.9
4	6.80 (s)	99.5	4		159.2		159.2
5		156.0	5		110.5		110.5
6	6.72 (d, 15.0)	129.6	6-OH	12.73 (s)	161.6	12.70 (s)	161.6
7	7.11 (dd, 15.0, 11.4)	131.9	7		204.4		204.3
8	7.25 (dd, 15.0, 11.4)	141.5	8	3.06 (m)	44.3	3.00 (dd,17.2, 8.7) 3.11 (dd,17.2, 3.0)	45.7
9	6.25 (d, 15.0)	128.2	9	4.21 (m)	67.4	4.40 (dqd, 8.7, 6.3, 3.0)	64.3
10		168.0	10	1.58 (m)	36.4	1.31 (d, 6.3)	22.5
2-Me	1.83 (s)	9.4	11	2.16 (overlap)	28.7		
3-OMe	3.92 (s)	57.3	12	5.45 (overlap)	130.6		
			13	5.50 (overlap)	125.6		
			14	1.66 (d, 4.9)	17.9		
			3-Me	2.21 (s)	15.6	2.21 (s)	15.6
			5-Me	2.13 (s)	7.4	2.13 (s)	7.4

Citrinsorbicillinol B (**2**): yellow oil; [*α*]25 D -26.5 (*c* 0.24, ACN); ECD (ACN) λ_max_ (Δ*ε*) 218 (−4.17), 297 (+1.55), 344 (−1.95) nm; UV (MeOH) (log *ε*) *λ*_max_ 216 (3.63), 249 (2.81), 286 (3.55), 331 (2.96) nm; IR (KBr) ν_max_/cm^−1^ 3,751, 3,689, 3,651, 2,931, 2,376, 1739, 1718, 1,621, 1,560, 1,523, 1,388, 1,305, 1,232, 1,161, 1,111, 1,022; HRESIMS *m/z* 301.1415 [M + Na]^+^ (calcd for C_16_H_22_O_4_Na^+^ 301.1416); ^1^H NMR and ^13^C NMR data, see Table 1.

Citrinsorbicillinol C (**3**): yellow powder; [*α*]25 D -20.4 (*c* 0.22, ACN); ECD (ACN) λ_max_ (Δε) 217 (−2.54), 284 (+1.70), 324 (−1.11) nm; UV (MeOH) (log *ε*) *λ*_max_ 217 (3.45), 249 (2.36), 385 (3.36), 330 (2.98) nm; IR (KBr) ν_max_/cm^−1^ 3,398, 2,927, 2,378, 1712, 1,678, 1,662, 1,625, 1,537, 1,519, 1,487, 1,452, 1,384, 1,230, 1,166, 1,026; HRESIMS *m/z* 247.0946 [M + Na]^+^ (calcd for C_12_H_16_O_4_Na^+^ 247.0955); ^1^H NMR and ^13^C NMR data, see Table 1.

### 2.5 Conformational optimization

The initial conformational analysis of compounds **1**–**3** was carried out using the SPARTAN’ 14 program, which employed the Monte Carlo searching algorithm with the MMFF94 molecular mechanics force field (Halgren, 1999). This resulted in a set of relatively stable conformations within an energy range of 3 kcal/mol above the global minimum. The conformers with minimum energy from the force field were then further optimized using density functional theory (DFT) at the B3LYP/6-31G(d) level, as implemented in the Gaussian 16 program. To ensure the reliability of the optimized conformers, harmonic vibrational frequency calculations were performed, confirming the absence of imaginary frequencies. The conformers accounting for over 99% of the population were then subjected to subsequent calculations.

### 2.6 ECD calculations

The predominant conformers of compounds **2** and **3** were subjected to theoretical ECD calculations using time-dependent density functional theory (TDDFT) at the M062X/def2SVP level in acetonitrile, employing the conductor-like polarizable continuum model (CPCM) for the solvent model. For each conformer, 30 excited states were calculated. The energies, oscillator strengths, and rotational strengths of each conformer were computed using the Gaussian 16 program. The ECD spectra for each conformer were approximated using Gaussian distributions. Finally, the overall ECD spectrum was determined by summing the spectra of individual conformers, weighted according to their Boltzmann populations, using the SpecDis v1.71 program (Bruhn et al., 2013).

### 2.7 ^13^C NMR calculations

The NMR shielding constants were calculated using the gauge-independent atomic orbital (GIAO) method at the mPW1PW91-SCRF/6–31 + G(2d,p) level with the PCM solvent model in dimethyl sulfoxide (DMSO). The shielding constants obtained were converted into chemical shifts by referencing TMS at 0 ppm (*δ*_cal_ = *σ*_TMS_ − *σ*_cal_), where *σ*TMS was the shielding constant of TMS calculated at the same level (Willoughby et al., 2014). The DP4+ probabilities for each possible candidate were calculated using the Excel spreadsheet provided by Grimblat et al. (2015). For each possible candidate, we calculated the parameters *a* and *b* of the linear regression *δ*_cal_ = *aδ*_exp_ + *b*. Additionally, we computed the correlation coefficient, *R*^2^, the mean absolute error (MAE), which is defined as Σn|*δ*_cal_ − *δ*_exp_|/n, and the corrected mean absolute error (CMAE), which is defined as Σn|*δ*_corr_ − *δ*_exp_|/n, where *δ*_corr_ = (*δ*_cal_ − b)/a.

### 2.8 DPPH radical-scavenging activity

The antioxidant activity of compounds **1**–**6** was assessed based on their scavenging activity against the stable 1,1-diphenyl-2-picrylhydrazyl (DPPH) radical (Zhao et al., 2010).

Samples in methanol, with concentrations ranging from 2 to 200 μg/ml, were mixed with freshly prepared 0.1 mM DPPH in ethanol. Absorbance at 517 nm was measured after 30 min at room temperature. Anhydrous ethanol served as the blank control, and ascorbic acid was used as the positive control. The radical-scavenging activity was expressed as the percentage inhibition, calculated using the following formula: Inhibition (%) = [A_control_ − (_Asample_ − A_blank_)]/A_control_ × 100%. Three parallel experiments were performed.

### 2.9 Measurement of NO production

The inhibition of lipopolysaccharide-induced nitric oxide (NO) production in RAW 264.7 mouse macrophage cells was evaluated as follows (Kumar et al., 2024; Yin et al., 2021b): The cells were evaluated using 96-well plates (1 × 10^5^ cells/well) and allowed to adhere for 2 h at 37°C in 5% CO_2_ in air. Then, the cells were treated with 1 μg/ml lipopolysaccharide (LPS) for 24 h, with or without the test compound (5 μg/ml). DMSO was used as the solvent. All compounds were tested at a final concentration of 0.2% (v/v) in the cell-culture supernatant. The NO production was determined by measuring the accumulation of nitrite in the culture supernatant using the Griess reagent. The absorbance of the mixture was read at 540 nm using a microplate reader. L-NAME (*N*^ω^-nitro-L-arginine methyl ester) was used as the positive control. Data were presented based on three parallel experiments.

### 2.10 Data and statistical analysis

The data were expressed as mean ± SD. Error bars represented the three independent experiments. *p*-values were calculated using ordinary one-way ANOVA.

## 3 Results and discussion

### 3.1 Structural elucidation

Citrinsorbicillinol A (**1**) was isolated as a yellow amorphous powder with a purity of over 95%, as determined by the HPLC analysis (Supplementary Figure S26). The molecule formula C_12_H_12_O_5_ was established by HRESIMS (Supplementary Figure S5) at *m/z* 237.0799 [M + H]^+^ (calcd. for C_12_H_13_O_5_, 237.0763), corresponding to seven degrees of unsaturation. The ^1^H NMR data (Supplementary Figure S1 and Table 1) clearly exhibited the presence of 11 protons, including 2 singlet methyl groups at *δ*_H_ 1.83 and 3.92, a conjugated diene moiety with 4 olefinic protons at *δ*_H_ 6.25 (d, *J* = 15.0 Hz), 6.72 (d, *J* = 15.0 Hz), 7.11 (dd, *J* = 15.0, 11.4 Hz), and 7.25 (dd, *J* = 15.0, 11.4 Hz), and a singlet aromatic proton at *δ*_H_ 6.80. The ^13^C NMR data (Supplementary Figure S2 and Table 1) revealed a total of 12 resonances. These resonances were assigned with the help of HSQC data (Supplementary Figure S3) to two methyl groups at *δ*_C_ 9.4 and 57.3, five sp^2^ methine groups at *δ*_C_ 99.5, 128.2, 129.6, 131.9, and 141.5, and five non-protonated carbons at 102.8, 156.0, 163.6, 165.9, and 168.0.

**Figure 1:**
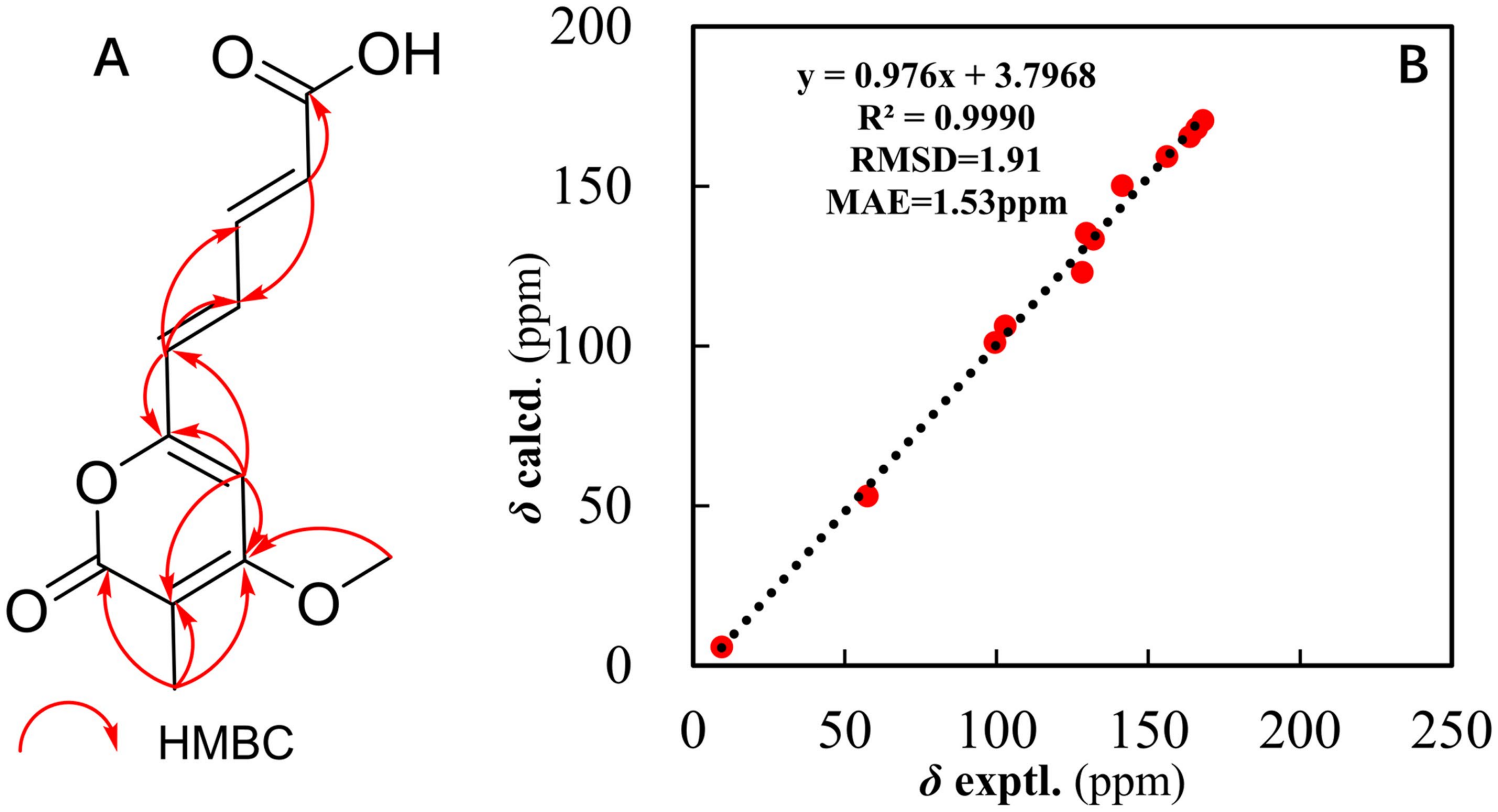
**(A)** Key HMBC correlations of compound **1**. **(B)** Regression analysis of the experimental versus calculated ^13^C NMR chemical shifts for structure ^#^**1**.

The structure of compound **1** was further elucidated through a comprehensive analysis of the 2D NMR data (Figure 1A). The key HMBC correlations (Supplementary Figure S4) from H-9 to C-10 and C-7; from H-8 to C-6, C-7, C-9, and C-10; and from H-6 to C-7 and C-8, along with the coupling constant of the related protons, support the construction of an *(E)*-penta-2,4-dienoic acid moiety. Moreover, the HMBC correlations from H_3_-2-Me to C-1, C-2, and C-3; from H_3_-3-OMe to C-3; from H-4 to C-2, C-3, C-5, and C-6; and from H-6 to C-4 and C-5, along with the chemical shift of C-5 at *δ*_C_ 156.0, suggested that the (*E*)-3-methoxy-2-methylpenta-2,4-dienoic acid moiety was connected to the *(E)*-penta-2,4-dienoic acid moiety via the oxygenated olefinic carbon at C-5. Considering the remaining unsaturation and the chemical shifts of C-1 at *δ*_C_ 163.6 and C-5 at *δ*_C_ 156.0, a lactone between C-1 and C-5 was deduced to establish the unsaturated six-membered lactone ring (Figure 1A).

**Figure 2:**
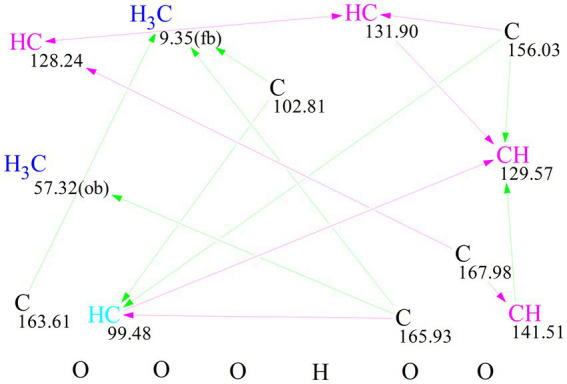
Molecular connectivity diagram (MCD) of **1**.

To further verify the structure, the computer-assisted structure elucidation (CASE) algorithm was employed. Specifically, ACD/Structure Elucidator (ACD/SE), an advanced CASE expert system, was utilized to automatically and efficiently determine the most probable structure based on chemical rules and common knowledge. In recent years, CASE analysis has increasingly been applied to structural revisions and identifications (Fan et al., 2022; Chen et al., 2024). The molecular formula and NMR data of compound **1**, which were analyzed using ACD/SE, led to the automatic generation of a molecular connectivity diagram (MCD), as shown in Figure 2. In the MCD (Figure 2), atom hybridization states are color-coded: sp3 is represented in blue, sp2 in violet, and undefined hybridization in black. The connecting lines are also color-coded to indicate different types of connectivity: green lines represent HMBC connectivity, and violet lines indicate non-standard connectivity for ^n^*J*_HH_ and ^n^*J*_CH_ (n > 3).

**Figure 3:**
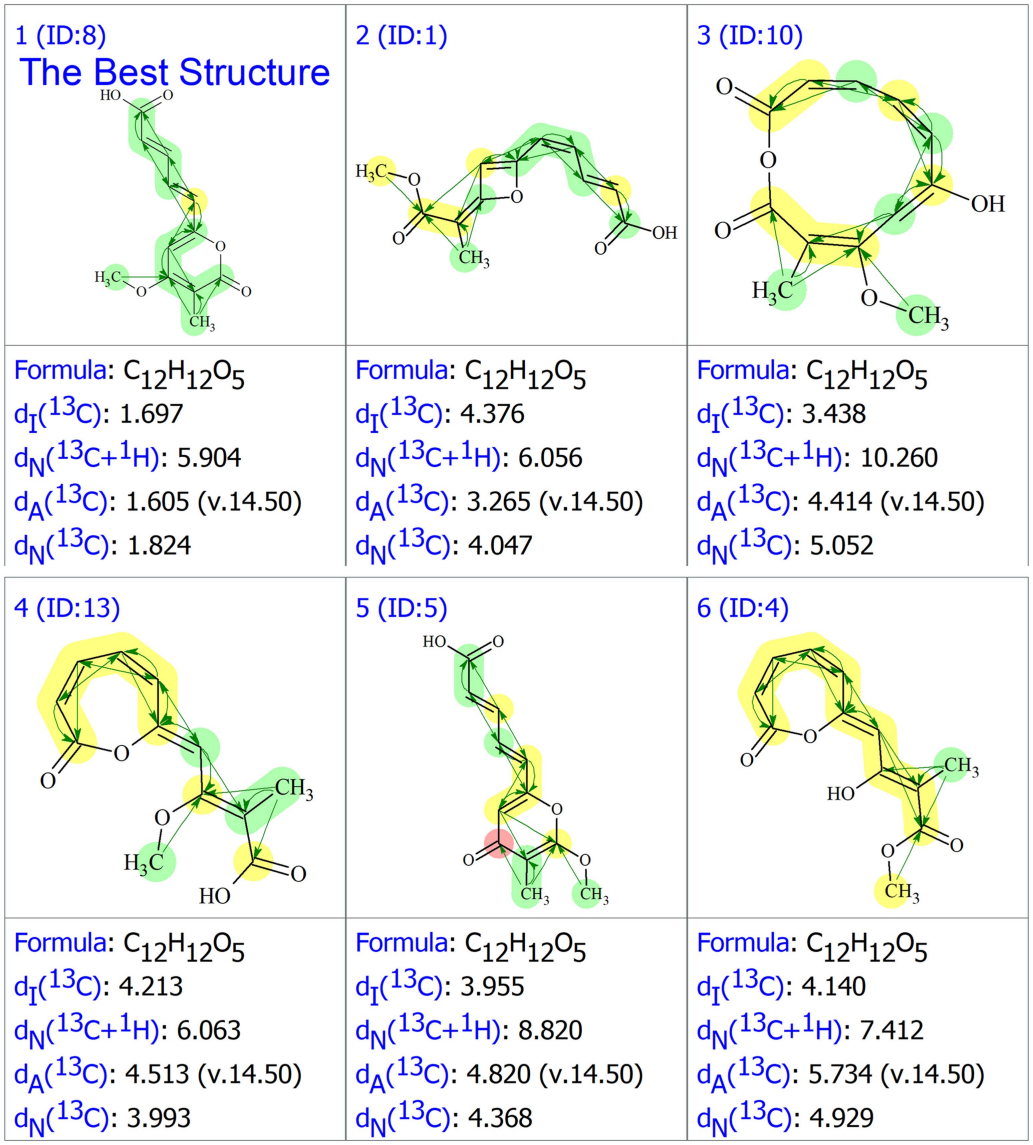
The generated six structures in the output file of ACD/SE.

For each candidate structure, ^1^H and ^13^C chemical shifts were predicted using three empirical methods provided by ACD/SE: HOSE (d_A_), the incremental approach (d_I_), and neural networks (d_N_). The structures that did not meet the threshold criteria of an average C deviation greater than 4 ppm or a maximum C deviation exceeding 20 ppm were discarded. The remaining structures were ranked based on the average deviation (d_A_) of the ^13^C chemical shifts between experimental and calculated values. The accuracy of the ^13^C chemical shift predictions was indicated by color-coded circles: green for the deviations ≤3 ppm and yellow for the deviations between 3 and 15 ppm. Ultimately, seven candidate structures were generated using ACD/SE from a total of 41,537 structures, and after removing the duplicates, six distinct structures remained, as shown in Figure 3. Among these, structure ^#^**1**, listed at the top of the output file from the ACD-Lab, exhibited the highest match factor (MF) value (d_A_ = 1.605, d_N_ = 1.824, and d_I_ = 1.697), which further confirmed its structure.

**Figure 4:**
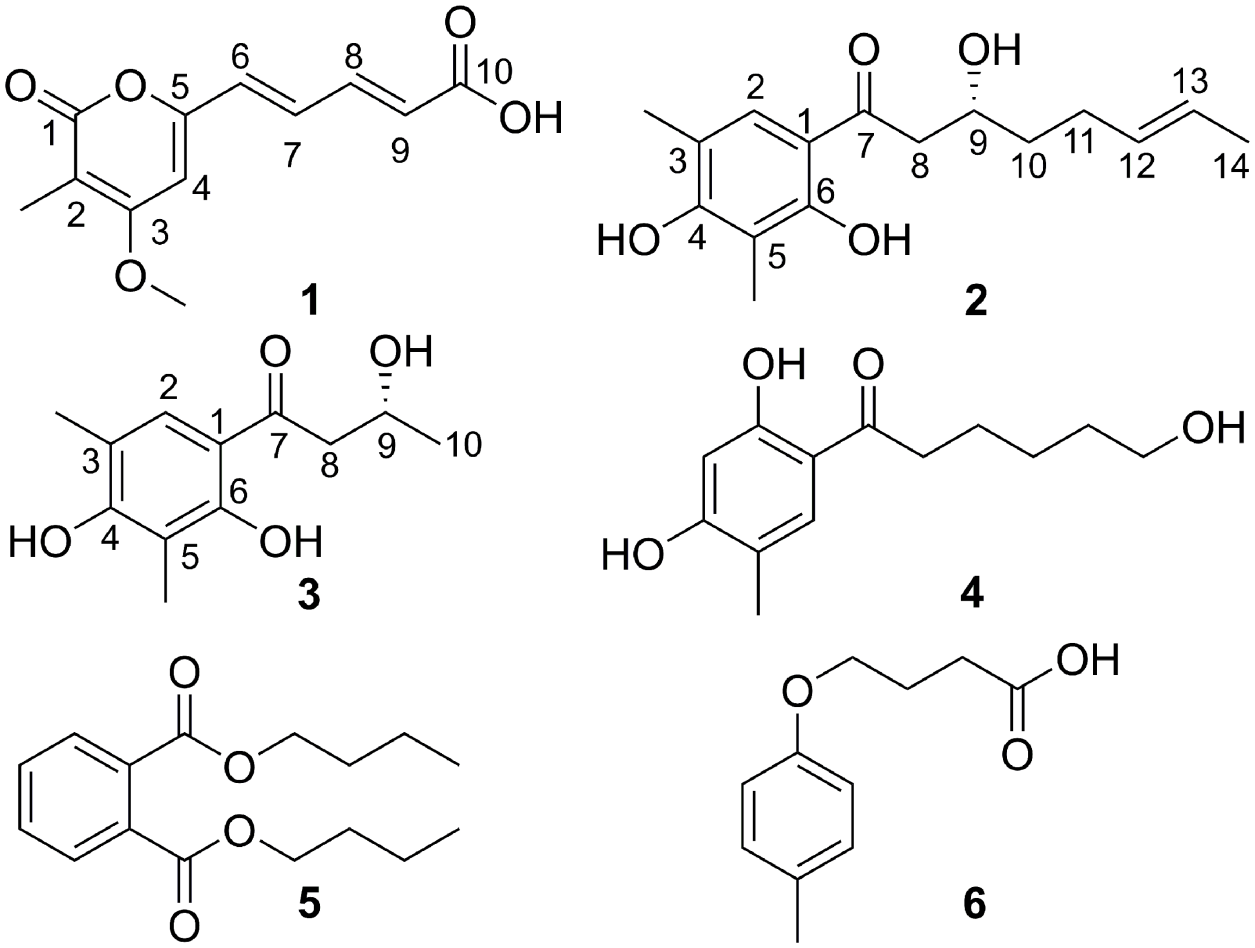
Structures of compounds **1**–**6**.

Furthermore, the ^13^C NMR calculation (Figure 1B) provided strong evidence for the assignment of structure ^#^**1**. This analysis was performed within the GIAO framework at the MPW1PW91/6–311 + G (2d, p) level, and geometries were optimized at the B3LYP/6-31G (d) level in chloroform. The correlation coefficient (R^2^) obtained from linear regression analysis between the calculated and experimental ^13^C NMR data for structure ^#^1 was 0.9990, and the mean absolute error (MAE) was 1.53 ppm. Thus, the structure of compound **1** was constructed (Figure 4).

**Figure 5:**
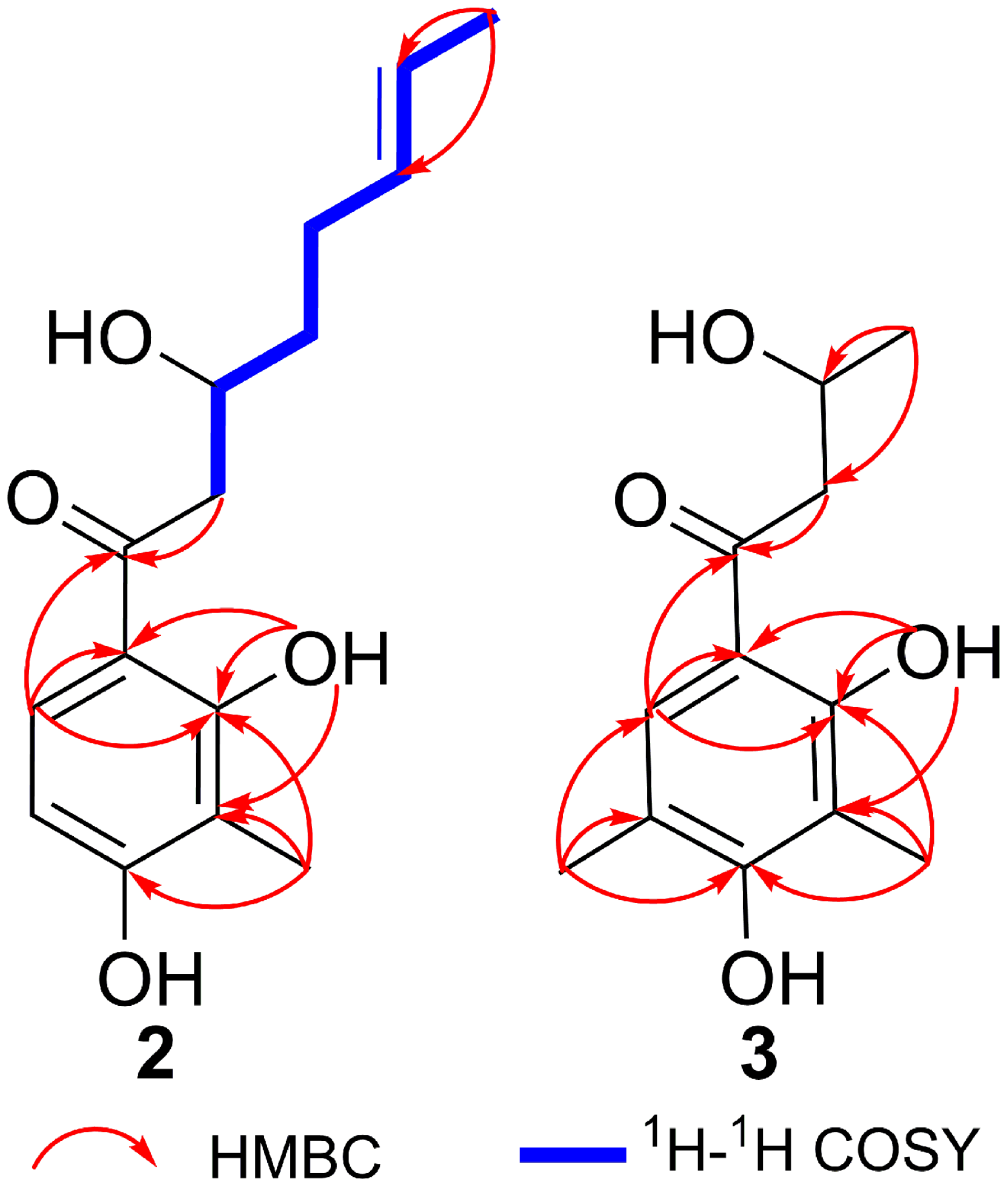
Key HMBC correlations of compounds **2** and **3**.

Citrinsorbicillinol B (**2**) was obtained as a yellow oil with a purity of over 95%, as determined by the HPLC analysis (Supplementary Figure S27). The molecular formula was determined as C_16_H_22_O_4_ based on a positive HRESIMS (Supplementary Figure S13) peak at *m/z* 301.1415 [M + Na]^+^ (calcd. for C_16_H_22_O_4_Na, 301.1416), which required six degrees of unsaturation. The ^1^H NMR data (Supplementary Figure S8 and Table 1) for compound **2** showed signals for two singlet methyl groups at *δ*_H_ 2.13 and 2.21, one doublet methyl group at *δ*_H_ 1.66 (d, *J* = 4.9 Hz), two olefinic protons at *δ*_H_ 5.50 and 5.45, one aromatic proton at *δ*_H_ 7.36 (s), and one typical hydroxyl proton at *δ*_H_ 12.73 (s). The ^13^C NMR and HSQC data (Supplementary Figures S9, S10 and Table 1) displayed 16 carbon resonances, accounting for three methyl groups at *δ*_C_ 7.4, 15.6, and 17.9, three sp^3^ methylene groups at *δ*_C_ 28.7, 36.4, and 44.3, three sp^2^ methine groups at *δ*_C_ 125.6, 129.2, and 130.6, one sp^3^ methine group at *δ*_C_ 67.4, and six non-protonated carbons at *δ*_C_ 110.5, 113.3, 114.9, 159.2, 161.6, and 204.4. In the 2D NMR spectra (Figure 5), the ^1^H-^1^H COSY correlations (Supplementary Figure S12) of H_3_-14/H-13/H-12/H_2_-11/H_2_-10/H-9/H_2_-8, along with the key HMBC correlations (Supplementary Figure S11) from H_3_-14 to C-12 and C-13; from H_2_-10 to C-11, C-12, C-8, and C-9; and from H_2_-8 to C-7, C-9, and C-10, supported the construction of an (*E*)-3-hydroxynon-6-en-1-one moiety. Then, a 2,4-dimethylbenzene-1,3-diol moiety was confirmed by the key HMBC correlations from H_3_-3-Me to C-2, C-3, and C-4; from H_3_-5-Me to C-4, C-5, and C-6; from H-2 to C-1 and C-6; and from OH-6 to C-1, C-5, and C-6. Moreover, the key HMBC correlations from H-2 to C-7 indicated that the (*E*)-3-hydroxynon-6-en-1-one moiety at C-7 was connected to the 2,4-dimethylbenzene-1,3-diol moiety at C-1 by a C-C single bond. Hence, the planar structure of compound **2** was established (Figure 4).

**Figure 6:**
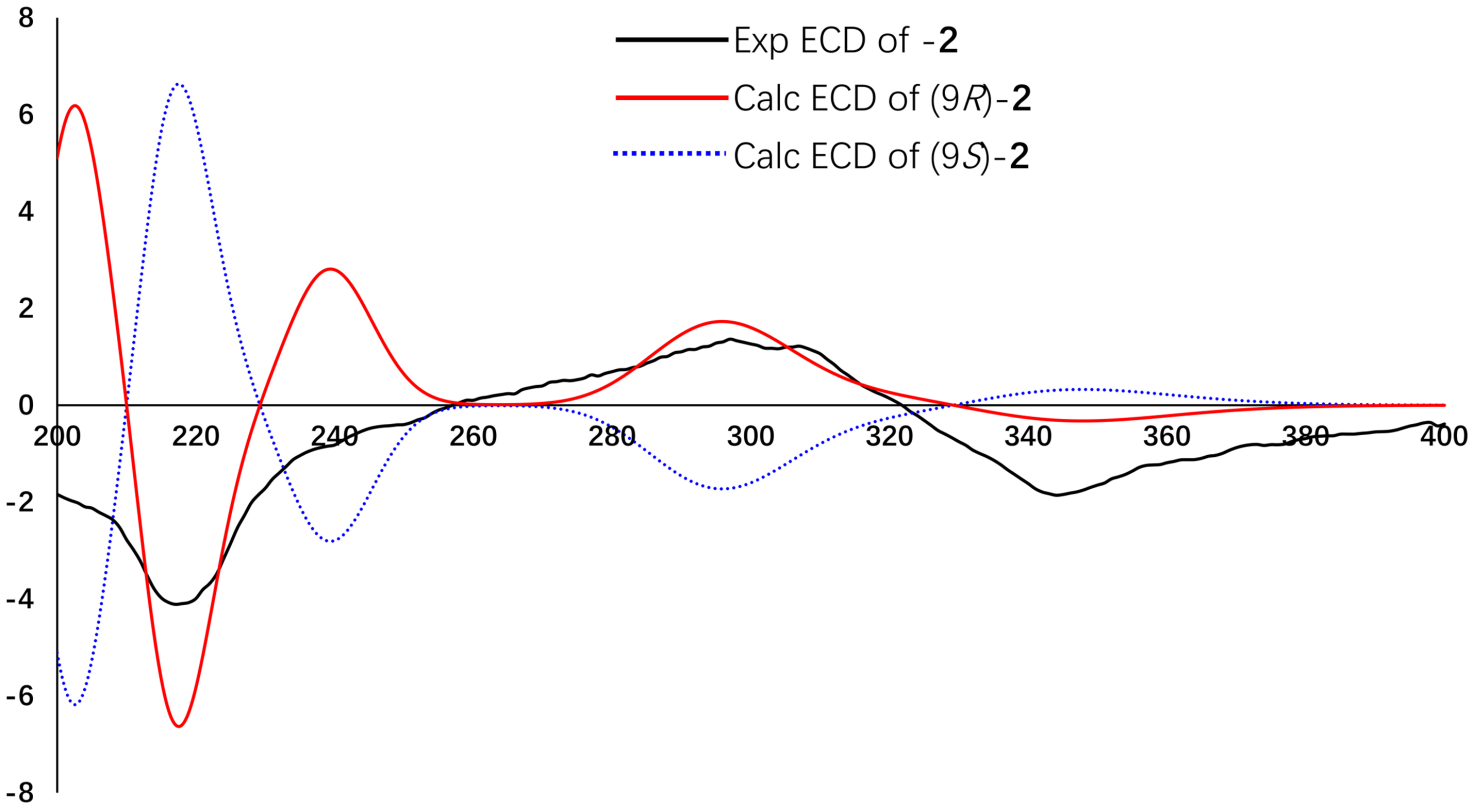
Experimental and calculated ECD spectra of compound **2**.

The absolute configuration of compound **2** was determined using TDDFT calculations of the ECD spectrum with the Gaussian 16 program. The ECD spectrum calculated for the 9*R* configuration was consistent with the experimental ECD spectrum (Figure 6). Hence, the absolute configuration of compound **2** was assigned as 9*R* (Figure 4).

**Figure 7:**
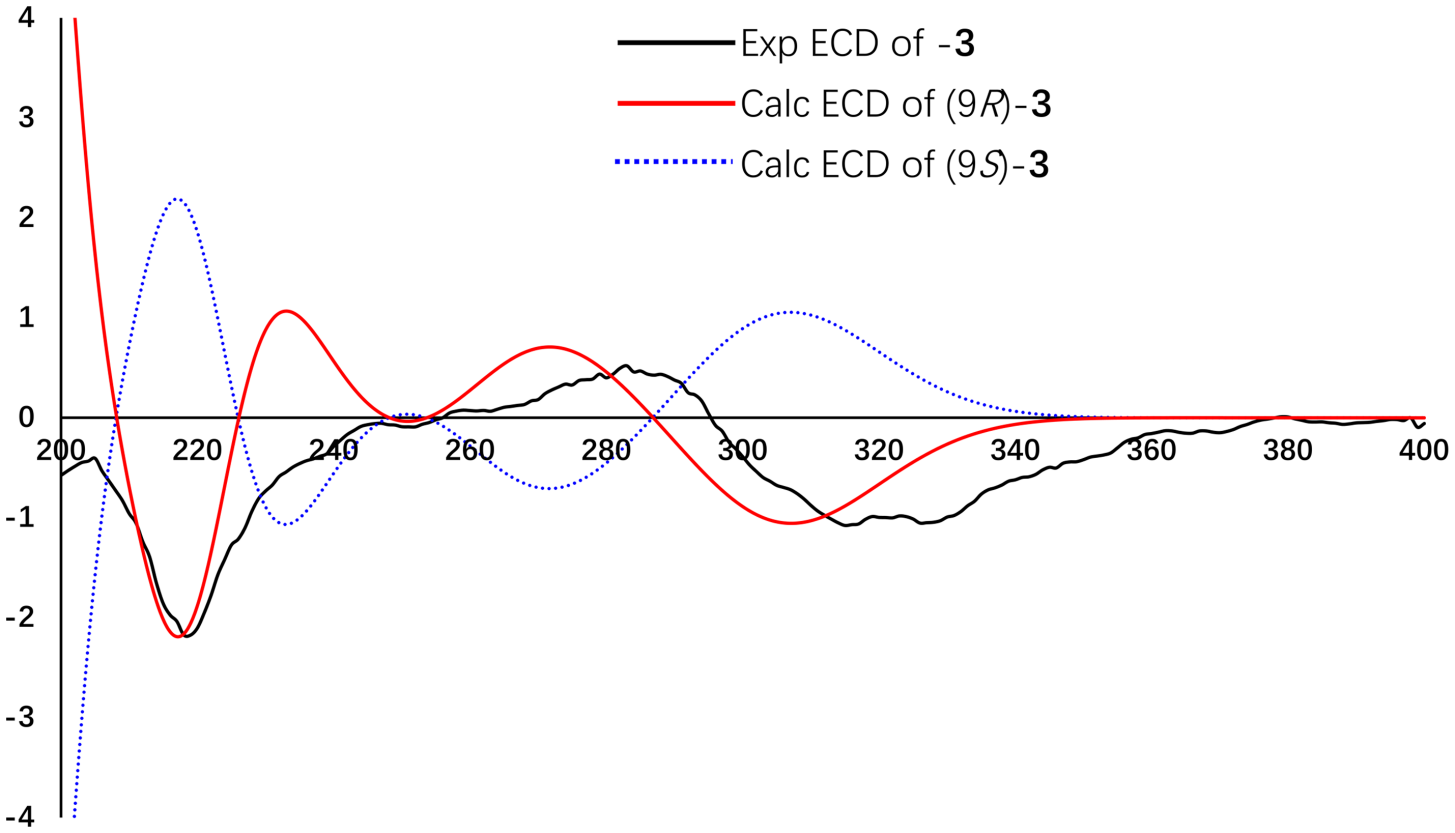
Experimental and calculated ECD spectra of compound **3**.

Citrinsorbicillinol C (**3**) was obtained as a yellow powder with a purity of over 95%, as determined by the HPLC analysis (Supplementary Figure S28). The molecular formula of compound **3** was established as C_12_H_16_O_4_ based on its HREIMS data (Supplementary Figure S21), indicating that it was the unit of C_4_H_6_ less than compound **2**. Detailed analysis of its NMR spectroscopic (Supplementary Figures S17, S18 and Figure 4) features implied that its chemical structure was very similar to that of compound **2**. The main difference was that compound **3** was missing two aliphatic methylene groups and two olefinic methine groups compared to compound **2**. Further analysis of the 2D NMR data (Supplementary Figures S19, S20 and Figure 5) showed that the side chain of compound **3** lacked an n-butene unit compared to that of compound **2**. This was supported by the key HMBC correlations from H_3_-3-Me to C-2, C-3, and C-4; from H_3_-5-Me to C-4, C-5, and C-6; from H_3_-10 to C-8 and C-9; from H_2_-8 to C-1 and C-7; from H-2 to C-1 and C-7; and from OH-6 to C-1, C-5, and C-6. Finally, the absolute configuration was established as 9*R* by comparing the experimental ECD spectrum with the calculated one (Figure 7).

In addition, three known compounds were finally characterized as trichosorbicillin G (**4**) (Zhang et al., 2019), dibutyl phthalate (**5**) (Li et al., 2009), and 3-(4-Methoxyphenyl) propanoic acid (**6**) (Zou et al., 2021), by comparing their spectroscopic data with the literature.

### 3.2 Biological activities

The isolated compounds **1**–**6** were tested for their antioxidant properties using the DPPH assay (Table 2). As a result, compounds **1**–**4** exhibited radical scavenging activity, with IC_50_ values ranging from 27.8 to 89.6 μM. Notably, compounds **2** and **3**, with IC_50_ values of 27.8 and 31.2 μM, respectively, exhibited significant activity comparable to that of ascorbic acid (IC_50_ = 39.4 μM). Structurally, compounds **2** and **3** each possessed two identical phenolic hydroxyl groups and one similar alcohol hydroxyl group. Compound **4** had a similar structure but with different substituents on the benzene ring and a different alcohol hydroxyl group. In contrast, compound **1** contained a carboxylic acid group with a long conjugated system. These results indicated that the number and position of phenolic and alcohol hydroxyl groups are crucial for antioxidant activity, with the substituents on the benzene ring also playing a significant role. Furthermore, the presence of a long conjugated carboxylic acid group could be a potent fragment contributing to antioxidant activity.

**Table 2:** The DPPH radical-scavenging activity and inhibitory effects on the NO Production of compounds **1**–**6**.

Compounds	IC_50_ (μM)	
	DPPH	NO
1	89.6 ± 1.1	52.7 ± 0.8
2	27.8 ± 0.5	>100
3	31.2 ± 0.4	>100
4	51.2 ± 1.0	>100
5	> 100	>100
6	> 100	>100
Ascorbic acid	39.4 ± 0.9	-
L-NAME*^a^*	-	48.6 ± 0.6

*p < 0.05, compared to the control group.*

In addition, all compounds were tested for their inhibitory activities toward nitric oxide (NO) production in lipopolysaccharide (LPS)-induced RAW 264.7 macrophages (Table 2). None of the compounds showed significant cytotoxicity at the concentrations of 50 μM for the inhibition of the NO production. Among them, only compound **1** showed moderate inhibitory activity, with an IC_50_ value of 52.7 μM.

## 4 Conclusion

In this study, three new polyketides **1**–**3** and three known compounds **4**–**6** were isolated and identified from the endophytic fungus *Trichoderma citrinoviride* associated with *Coptis chinensis*. The biological assays demonstrated that compounds **1**–**4** exhibited notable antioxidant activity, highlighting their potential as effective agents in mitigating oxidative damage. Particularly, compounds **2** and **3** showed significant radical scavenging capabilities, comparable to the positive control, ascorbic acid. In addition, compound **1** exhibited promising anti-inflammatory effects by inhibiting the nitric oxide production. Overall, these identified compounds not only contribute to the expanding collection of bioactive natural products but also offer promising avenues for the development of new antioxidant and anti-inflammatory agents. Future work should focus on further exploring the mechanisms underlying these biological activities and assessing the efficacy of these compounds in more complex biological systems.

## Data Availability

The original contributions presented in the study are included in the article/Supplementary material, further inquiries can be directed to the corresponding authors.

## References

[ref1] Barzkar N. Sukhikh S. Babich O. (2024). Study of marine microorganism metabolites: new resources for bioactive natural products. Front. Microbiol. 14:1285902. doi: 10.3389/fmicb.2023.1285902, PMID: 38260902 PMC10800913

[ref2] Bruhn T. Schaumlöffel A. Hemberger Y. Bringmann G. (2013). SpecDis: quantifying the comparison of calculated and experimental electronic circular dichroism spectra. Chirality 25, 243–249. doi: 10.1002/chir.22138, PMID: 23532998

[ref3] Cai J. Zhou X. Wang B. Zhang X. Luo M. Huang L.. (2024). Bioactive polyketides and meroterpenoids from the mangrove-derived fungus *Talaromyces flavus* TGGP35. Front. Microbiol. 15:1342843. doi: 10.3389/fmicb.2024.1342843, PMID: 38362503 PMC10867163

[ref4] Chen S. Guo H. Wu Z. Wu Q. Jiang M. Li H.. (2022). Targeted discovery of sorbicillinoid pgments with anti-inflammatory activity from the sponge-derived fungus *Stagonospora* sp. SYSU-MS7888 using the PMG strategy. J. Agric. Food Chem. 70, 15116–15125. doi: 10.1021/acs.jafc.2c05940, PMID: 36410725

[ref5] Chen D. Xu X. Yang Y. Meng H. Xu M. Dong L.. (2024). Discovery of cadinane-type sesquiterpenoids from the infectedstems of *Hibiscus tiliaceus* as potential agrochemical fungicides. J. Agric. Food Chem. 72, 4089–4099. doi: 10.1021/acs.jafc.3c08508, PMID: 38353561

[ref6] Fan K. Zhang L. C. Hu W. Y. Deng S. Y. Wu H. Tan B. Y.. (2022). Tabernaecorymine a, an 18-normonoterpenoid indole alkaloid with antibacterial activity from *Tabernaemontana corymbosa*. Fitoterapia 157:105129. doi: 10.1016/j.fitote.2022.105129, PMID: 35051555

[ref7] Grimblat N. Zanardi M. M. Sarotti A. M. (2015). Beyond DP4: an improved probability for the stereochemical assignment of isomeric compounds using quantum chemical calculations of NMR shifts. J. Organomet. Chem. 80, 12526–12534. doi: 10.1021/acs.joc.5b02396, PMID: 26580165

[ref8] Halgren T. A. (1999). MMFF VI. MMFF94s option for energy minimization studies. J. Comput. Chem. 20, 720–729. doi: 10.1002/(SICI)1096-987X(199905)20:7<720::AID-JCC7>3.0.CO;2-X, PMID: 34376030

[ref9] Kahlert L. Bassiony E. F. Cox R. J. Skellam E. J. (2020). Diels-alder reactions during the biosynthesis of sorbicillinoids. Angew. Chem. Int. Ed. 59, 5816–5822. doi: 10.1002/anie.201915486, PMID: 31943627 PMC7154774

[ref10] Kumar P. Wallis M. Zhou X. Li F. Holland D. C. Reddell P.. (2024). Triplinones A-H: anti-inflammatory arylalkenyl α,β-unsaturated-δ-lactones isolated from the leaves of Australian rainforest plant *Cryptocarya triplinervis* (Lauraceae). J. Nat. Prod. 87, 1817–1825. doi: 10.1021/acs.jnatprod.4c00454, PMID: 38964296

[ref11] Li J. T. Yin B. L. Liu Y. Wang L. Q. Chen Y. G. (2009). Mono-aromatic constituents of *Dendrobium longicornu*. Chem. Nat. Compd. 45, 234–236. doi: 10.1007/s10600-009-9293-2

[ref12] Peng X. P. Li G. Wang L. M. Wang Q. Wang C. Ji L. X.. (2022). Structurally various sorbicillinoids from an endophytic fungus *Acremonium citrinum* SS-g13. Front. Microbiol. 13:800626. doi: 10.3389/fmicb.2022.800626, PMID: 35418970 PMC8997241

[ref13] Rehman S. U. Yang L. J. Zhang Y. H. Wu J. S. Shi T. Haider W.. (2020). Sorbicillinoid derivatives from sponge-derived fungus *Trichoderma reesei* (HN-2016-018). Front. Microbiol. 11:1334. doi: 10.3389/fmicb.2020.01334, PMID: 32655528 PMC7325520

[ref14] Sib A. Gulder T. A. M. (2017). Stereoselective total synthesis of bisorbicillinoid natural products by enzymatic oxidative dearomatization/dimerization. Angew. Chem. Int. Ed. 56, 12888–12891. doi: 10.1002/anie.201705976, PMID: 28771960

[ref15] Wang Y. J. Chen X. Yin Y. Zhou W. Zhou P. F. Zeng L. G.. (2024). Hedscandines A-C, three undescribed indole alkaloids from *Hedyotis scandens* with their anti-MRSA activity. Phytochemistry 219:113988. doi: 10.1016/j.phytochem.2024.113988, PMID: 38224846

[ref16] Wang Y. Li X. M. Yang S. Q. Zhang F. Z. Wang B. G. Li H. L.. (2022). Sesquiterpene and sorbicillinoid glycosides from the endophytic fungus *Trichoderma longibrachiatum* EN-586 derived from themarine red alga *Laurencia obtusa*. Mar. Drugs 20:177. doi: 10.3390/md20030177, PMID: 35323476 PMC8949086

[ref17] Wang F. Zhang M. Yuan M. Xia Z. Yang F. Zhang S.. (2023). A novel sorbicillinoid compound as a potent anti-inflammation agent through inducing NLRP3 protein degradation. Br. J. Pharmacol. 180, 1930–1948. doi: 10.1111/bph.16058, PMID: 36788033 PMC10330222

[ref18] Willoughby P. H. Jansma M. J. Hoye T. R. (2014). A guide to small-molecule structure assignment through computation of (^1^H and ^13^C) NMR chemical shifts. Nat. Protoc. 9, 643–660. doi: 10.1038/nprot.2014.042, PMID: 24556787

[ref19] Xie C. L. Zhang D. Lin T. He Z. H. Yan Q. X. Cai Q.. (2021). Antiproliferative sorbicillinoids from the deep-sea-derived *Penicillium allii-sativi*. Front. Microbiol. 11:636948. doi: 10.3389/fmicb.2020.636948, PMID: 33552036 PMC7858254

[ref20] Yin G. P. Gong M. Li Y. J. Zhang X. Zhu J. J. Hu C. H. (2021a). 14-membered resorcylic acid lactone derivatives with their anti-inflammatory from the fungus *Aspergillus* sp. ZJ-65. Fitoterapia 151:104884. doi: 10.1016/j.fitote.2021.104884, PMID: 33766742

[ref21] Yin G. P. Gong M. Xue G. M. Gong T. Cao X. Wang W.. (2021b). Penispidins A-C, aromatic sesquiterpenoids from *Penicillium virgatum* and their inhibitory effects on hepatic lipid accumulation. J. Nat. Prod. 84, 2623–2629. doi: 10.1021/acs.jnatprod.1c00185, PMID: 34610746

[ref22] Yin G. P. Li Y. J. Zhang L. Zeng L. G. Liu X. M. Hu C. H. (2024). Citrinsorbicillin a, a novel homotrimeric sorbicillinoid isolated by LC-MS-guided with cytotoxic activity from the fungus *Trichoderma citrinoviride* HT-9. Chin. Chem. Lett. 35:109035. doi: 10.1016/j.cclet.2023.109035, PMID: 39816934

[ref23] Ying Y. Lei P. Xu Y. Lin Y. Yang N. Han Y.. (2024). Secondary metabolites from *Penicillium* sp. HS-11, a fungal endophyte of *Huperzia serrata*. Fitoterapia 175:105943. doi: 10.1016/j.fitote.2024.105943, PMID: 38575090

[ref24] Yu X. Müller W. E. G. Frank M. Gao Y. Guo Z. Zou K.. (2024). Caryophyllene-type sesquiterpenes from the endophytic fungus *Pestalotiopsis lespedezae* through an OSMAC approach. Front. Microbiol. 14:1248896. doi: 10.3389/fmicb.2023.1248896, PMID: 38274753 PMC10808731

[ref25] Zhang P. Deng Y. Lin X. Chen B. Li J. Liu H.. (2019). Anti-inflammatory mono- and dimeric sorbicillinoids from the marine-derived fungus *Trichoderma reesei* 4670. J. Nat. Prod. 82, 947–957. doi: 10.1021/acs.jnatprod.8b01029, PMID: 30920218

[ref26] Zhang Y. Zhang J. Du Q. Wu X. M. Chen Y. Tan R. X. (2024a). Citrisorbicillinol, an undescribed hybrid sorbicillinoid with osteogenic activity from *Penicillium citrinum* ZY-2. Fitoterapia 173:105836. doi: 10.1016/j.fitote.2024.105836, PMID: 38286315

[ref27] Zhang Y. Zhang Y. Li G. Dong K. Wang J. Xiao S.. (2024b). Anti-inflammatory monomeric sorbicillinoids from the marine-fish-derived fungus *Trichoderma* sp. G13. Fitoterapia 175:105963. doi: 10.1016/j.fitote.2024.105963, PMID: 38631598

[ref28] Zhang X. H. Zhang D. J. Liu J. L. Pan H. Y. Qin J. C. Zhang Y. H. (2018). Antifungal effects of volatile organic compounds from the endophytic fungus *Cryptosporiopsis ericae* cc-HG-7 isolated from *Coptis chinensis* Franch. Biocontrol Sci. Tech. 28, 496–508. doi: 10.1080/09583157.2018.1460744

[ref29] Zhao Y. Liu J. P. Lu D. Li P. Y. Zhang L. X. (2010). A new antioxidant xanthone from the pericarp of *Garcinia mangostana* Linn. Nat. Prod. Res. 24, 1664–1670. doi: 10.1080/14786419.2010.499539, PMID: 20954095

[ref30] Zhao D. L. Wang H. S. Gao L. W. Zhang P. (2022). Tennessenoid A, an unprecedented steroid-sorbicillinoid adduct from the marine-derived endophyte of *Aspergillus* sp. strain 1022LEF. Front. Mar. Sci. 9:923128. doi: 10.3389/fmars.2022.923128, PMID: 39807381

[ref31] Zou M. Wang R. Yin Q. Liu L. (2021). Bioassay-guided isolation and identification of anti-alzheimer’s active compounds fro*m Spiranthes sinensis* (Pers.) Ames. Med. Chem. Res. 30, 1849–1855. doi: 10.1007/s00044-021-02777-8

