# Supplementary material for: Bioactive sorbicillinoid derivatives from an endophytic fungus Trichoderma citrinoviride
Source: Front Microbiol. 2025 Jan 28;16:1485032. doi: 10.3389/fmicb.2025.1485032 (PMC11811624; doi:10.3389/fmicb.2025.1485032)

**Supporting Information**

Bioactive Sorbicillinoid Derivatives from an Endophytic Fungus *Trichoderma citrinoviride*

Yan-Ping Xia^1^, Yan Xie^2^, Li Rao^3*^, Guo-Ping Yin^4*^

^1^Department of Pharmacy, Nanan People’s Hospital of Chongqing, Chongqing, China

^2^Department of Pharmacy, Zhengzhou Shuqing Medical College, Zhengzhou, China

^3^Chongqing Key Laboratory of High Active Traditional Chinese Drug Delivery System, Chongqing Medical and Pharmaceutical College, Chongqing, China

^4^Engineering Research Center of Coptis Development and Utilization (Ministry of Education), College of Pharmaceutical Sciences and Chinese Medicine, Southwest University, Chongqing, China

*** Correspondence:**Li Rao (raoli0728@163.com); Guo-Ping Yin (yinguoping16@swu.edu.cn)

Keywords: sorbicillinoid, fungus, *Trichoderma*, antioxidant, anti-inflammatory

**List of supporting information**

[Figure S1. ^1^H NMR spectrum of compound 1 (400 MHz, DMSO-d_6_). 3](#_Toc186466844)

[Figure S2. ^13^C NMR spectrum of compound 1 (100 MHz, DMSO-d_6_) 3](#_Toc186466845)

[Figure S3. HSQC spectrum of compound 1 (DMSO-d_6_) 4](#_Toc186466846)

[Figure S4. HMBC spectrum of compound 1 (DMSO-d_6_) 4](#_Toc186466847)

[Figure S5. HRESIMS spectrum of compound 1 5](#_Toc186466848)

[Figure S6. UV spectrum of compound 1 5](#_Toc186466849)

[Figure S7. IR spectrum of compound 1 6](#_Toc186466850)

[Figure S8. ^1^H NMR spectrum of compound 2 (400 MHz, CDCl_3_). 6](#_Toc186466851)

[Figure S9. ^13^C NMR spectrum of compound 2 (100 MHz, CDCl_3_) 7](#_Toc186466852)

[Figure S10. HSQC spectrum of compound 2 (CDCl_3_) 7](#_Toc186466853)

[Figure S11. HMBC spectrum of compound 2 (CDCl_3_) 8](#_Toc186466854)

[Figure S12. ^1^H-^1^H COSY spectrum of compound 2 (CDCl_3_) 8](#_Toc186466855)

[Figure S13. HRESIMS spectrum of compound 2 9](#_Toc186466856)

[Figure S14. UV spectrum of compound 2 9](#_Toc186466857)

[Figure S15. IR spectrum of compound 2 10](#_Toc186466858)

[Figure S16. Experimental and computational ECD spectra of compound 2 10](#_Toc186466859)

[Figure S17. ^1^H NMR spectrum of compound 3 (400 MHz, CDCl_3_). 11](#_Toc186466860)

[Figure S18. ^13^C NMR spectrum of compound 3 (100 MHz, CDCl_3_) 11](#_Toc186466861)

[Figure S19. HSQC spectrum of compound 3 (CDCl_3_) 12](#_Toc186466862)

[Figure S20. HMBC spectrum of compound 3 (CDCl_3_) 12](#_Toc186466863)

[Figure S21. HRESIMS spectrum of compound 3 13](#_Toc186466864)

[Figure S22. UV spectrum of compound 3 13](#_Toc186466865)

[Figure S23. IR spectrum of compound 3 14](#_Toc186466866)

[Figure S24. Experimental and computational ECD spectra of compound 3 14](#_Toc186466867)

[Figure S25. Generated structures from ACD-Labs. 15](#_Toc186466868)

[Figure S26 HPLC chromatogram of compound 1. 15](#_Toc186466869)

[Figure S27 HPLC chromatogram of compound 2. 15](#_Toc186466870)

[Figure S28 HPLC chromatogram of compound 3. 16](#_Toc186466871)

[Figure S29. Colony morphology of Trichoderma citrinoviride on PDA after 5 days of growth 16](#_Toc186466872)

[Figure S30. Microscopic image of Trichoderma citrinoviride colony morphology on PDA After 3 days (magnified 40x). 16](#_Toc186466873)

# Figure S1. ^1^H NMR spectrum of compound 1 (400 MHz, DMSO-*d*_6_).


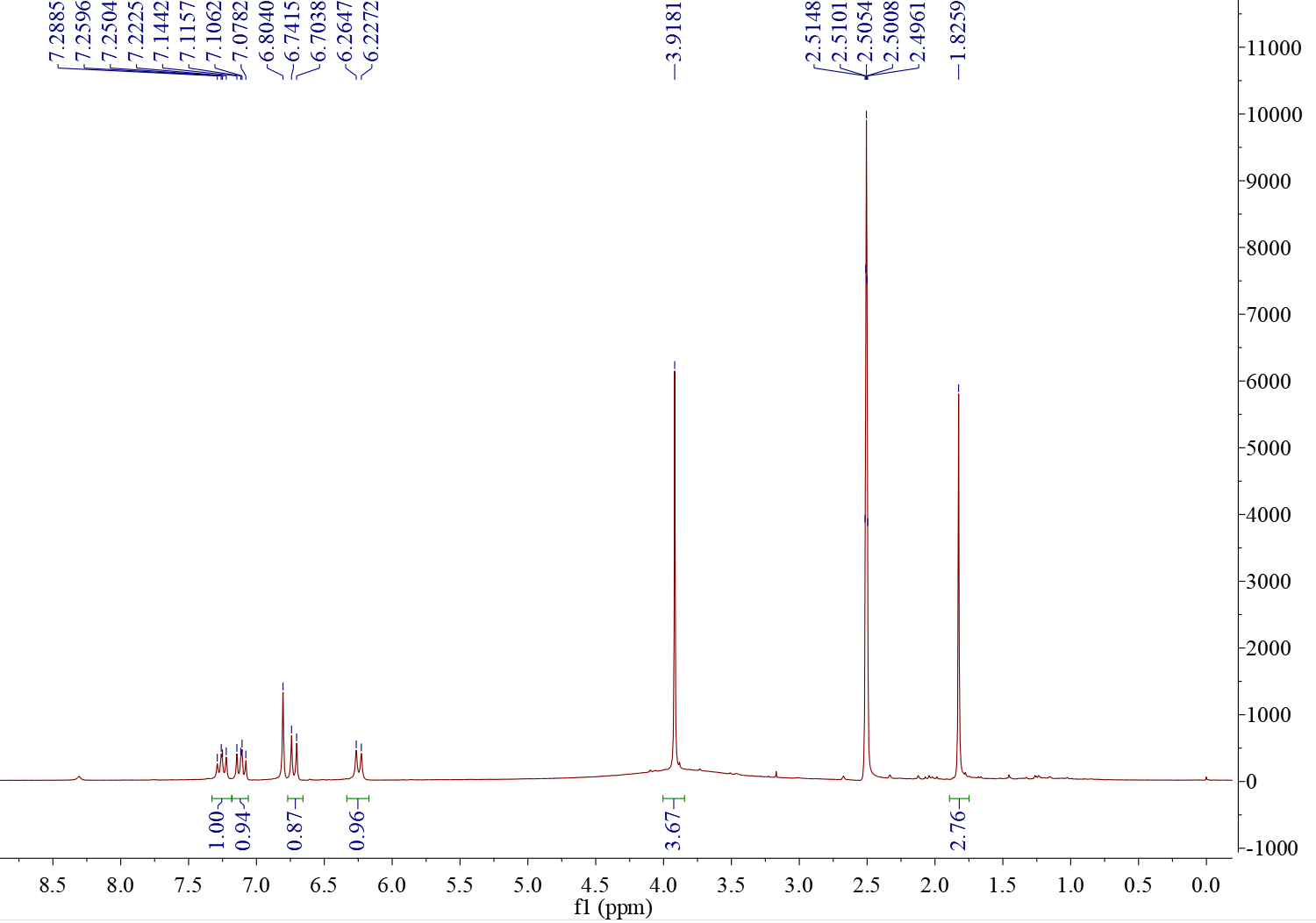


# Figure S2. ^13^C NMR spectrum of compound 1 (100 MHz, DMSO-*d*_6_)


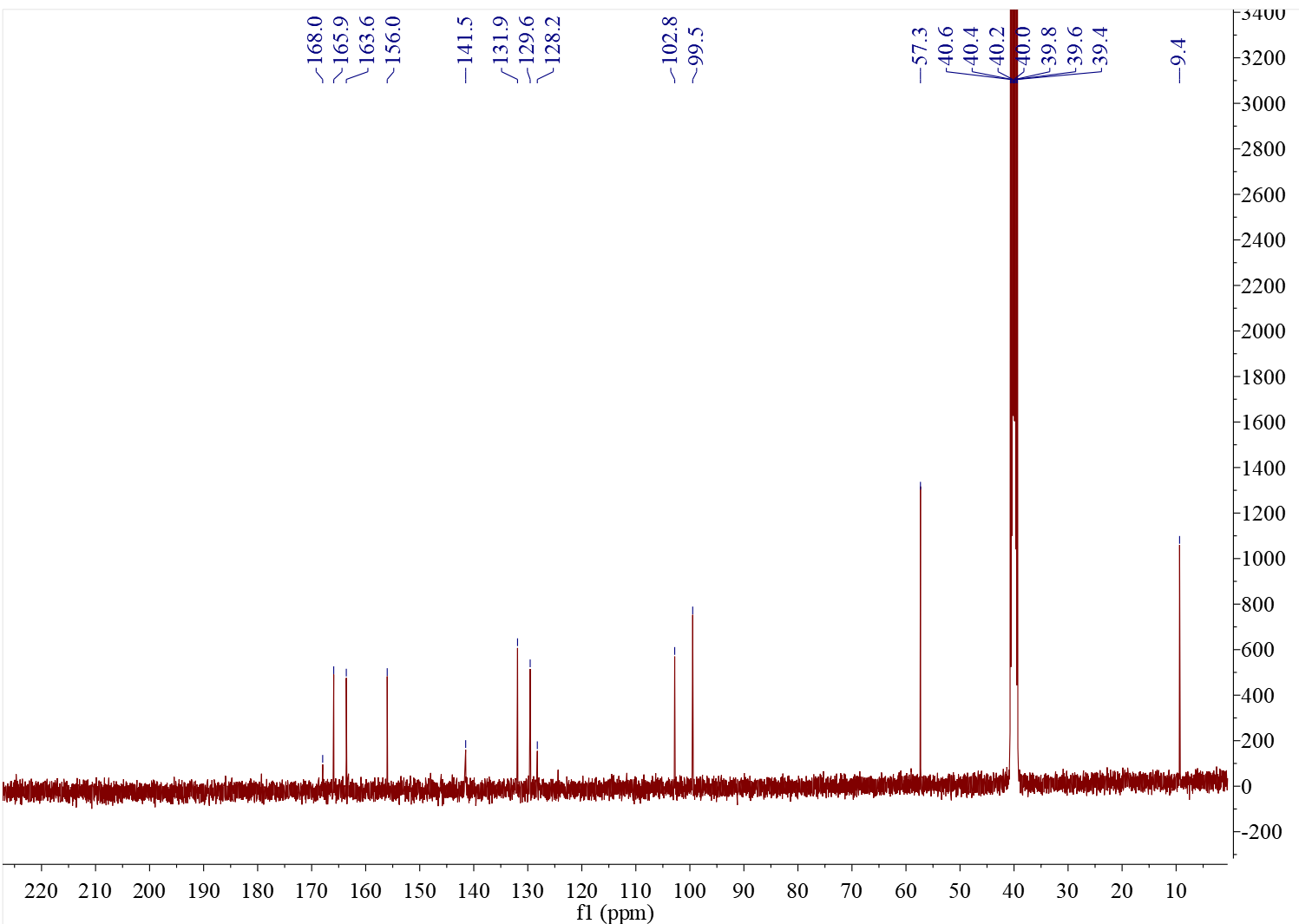


# Figure S3. HSQC spectrum of compound 1 (DMSO-*d*_6_)


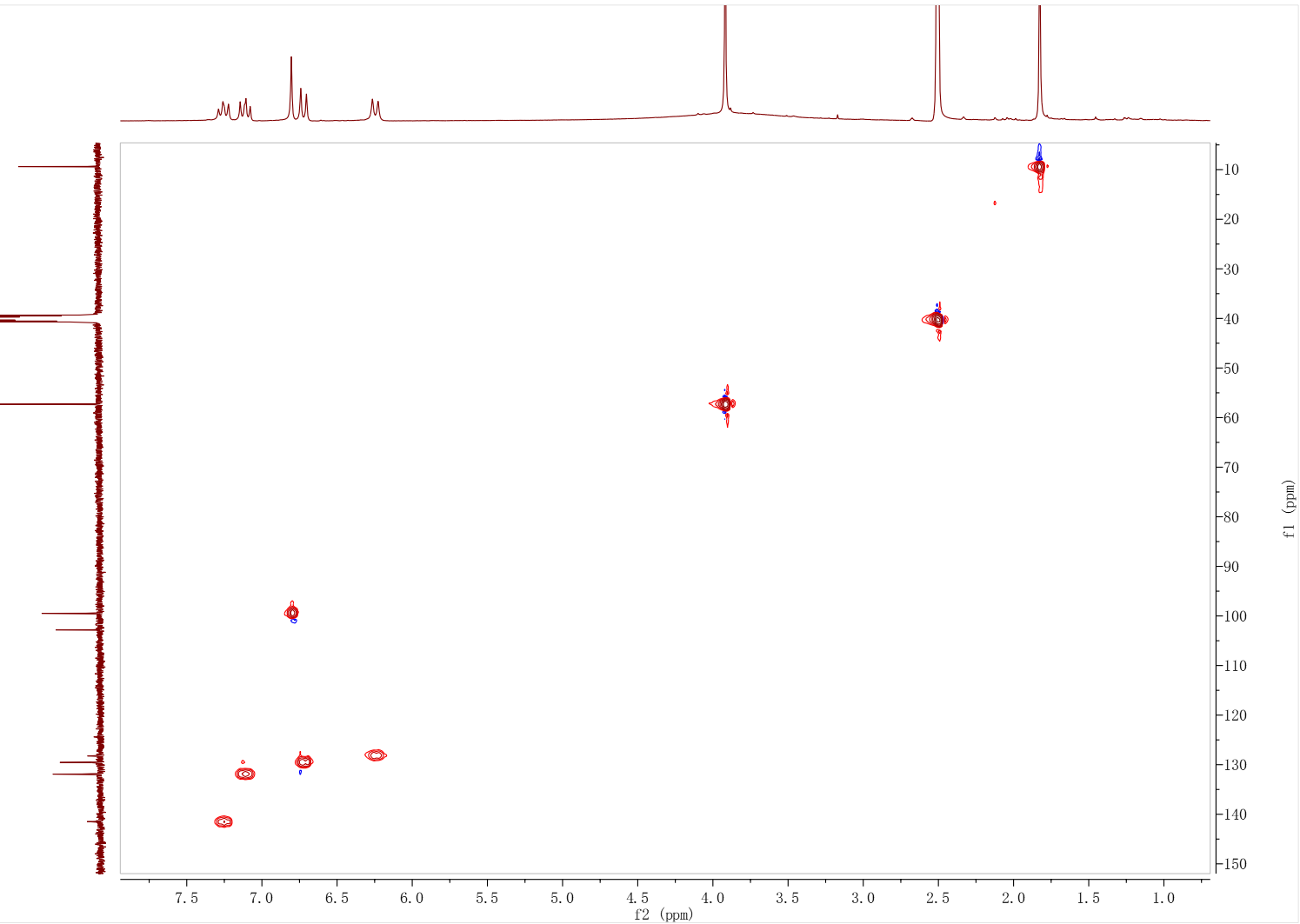


# Figure S4. HMBC spectrum of compound 1 (DMSO-*d*_6_)


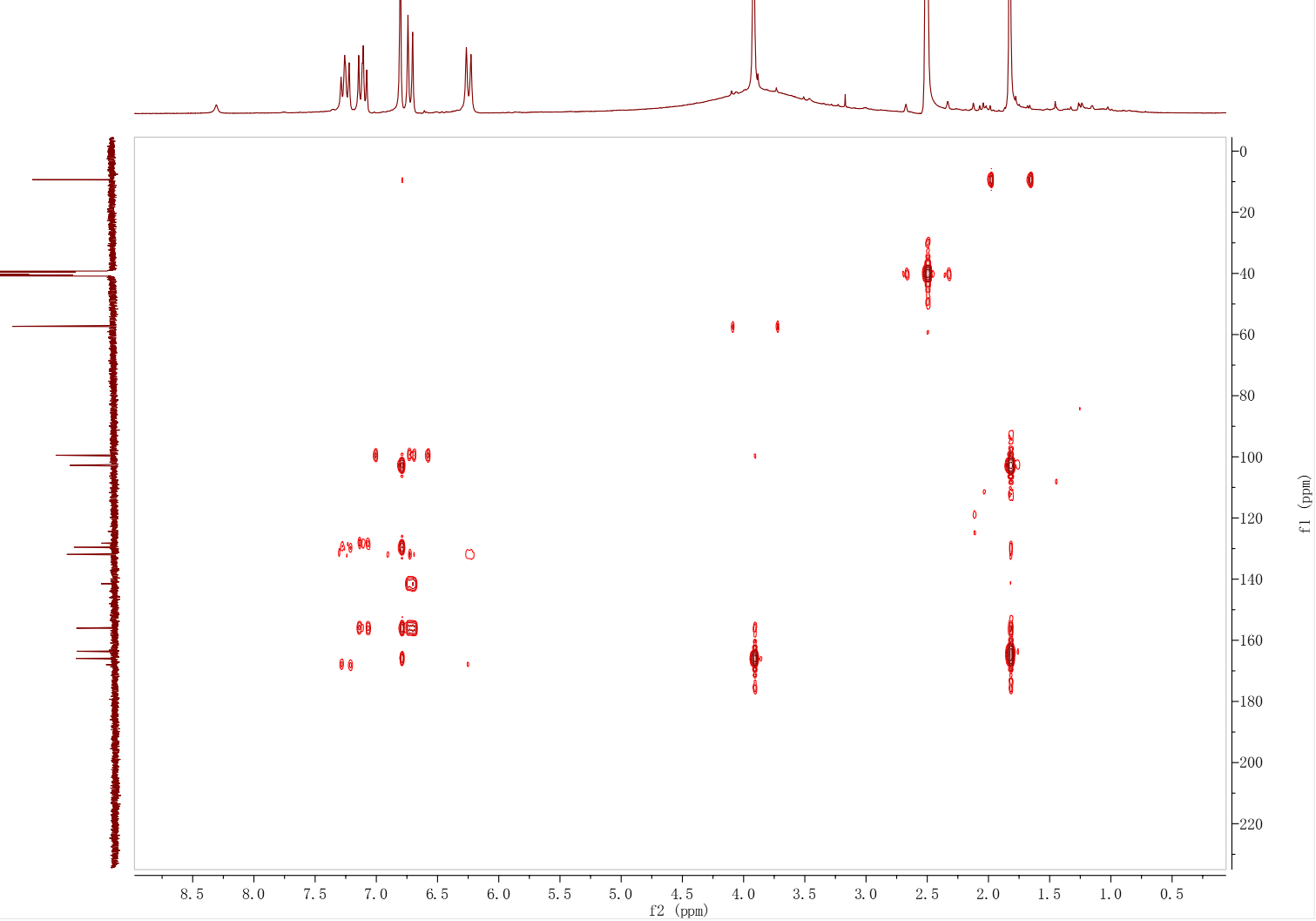


# Figure S5. HRESIMS spectrum of compound 1


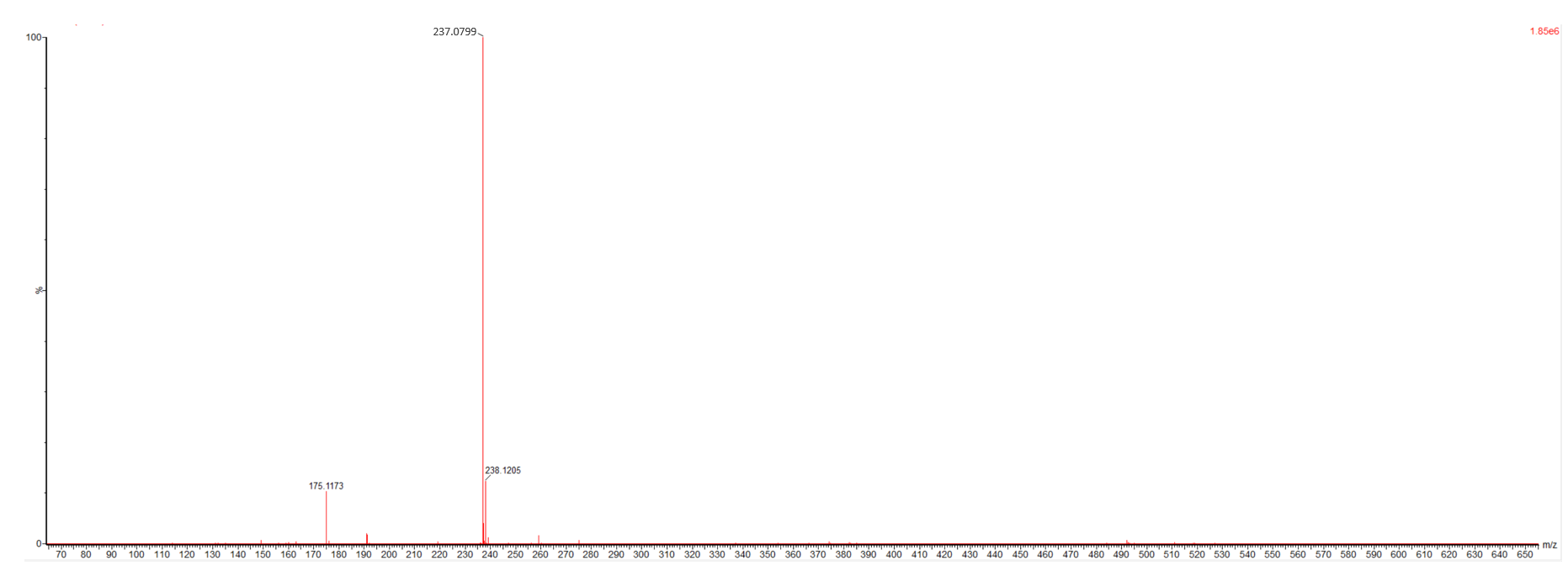


# Figure S6. UV spectrum of compound 1


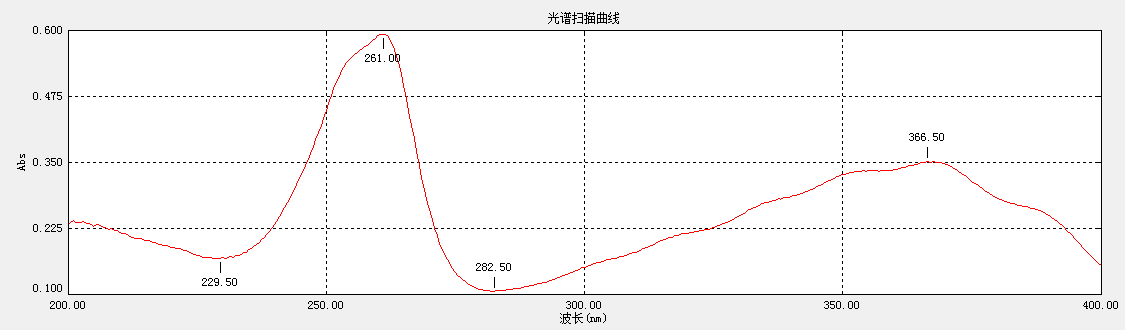


# Figure S7. IR spectrum of compound 1


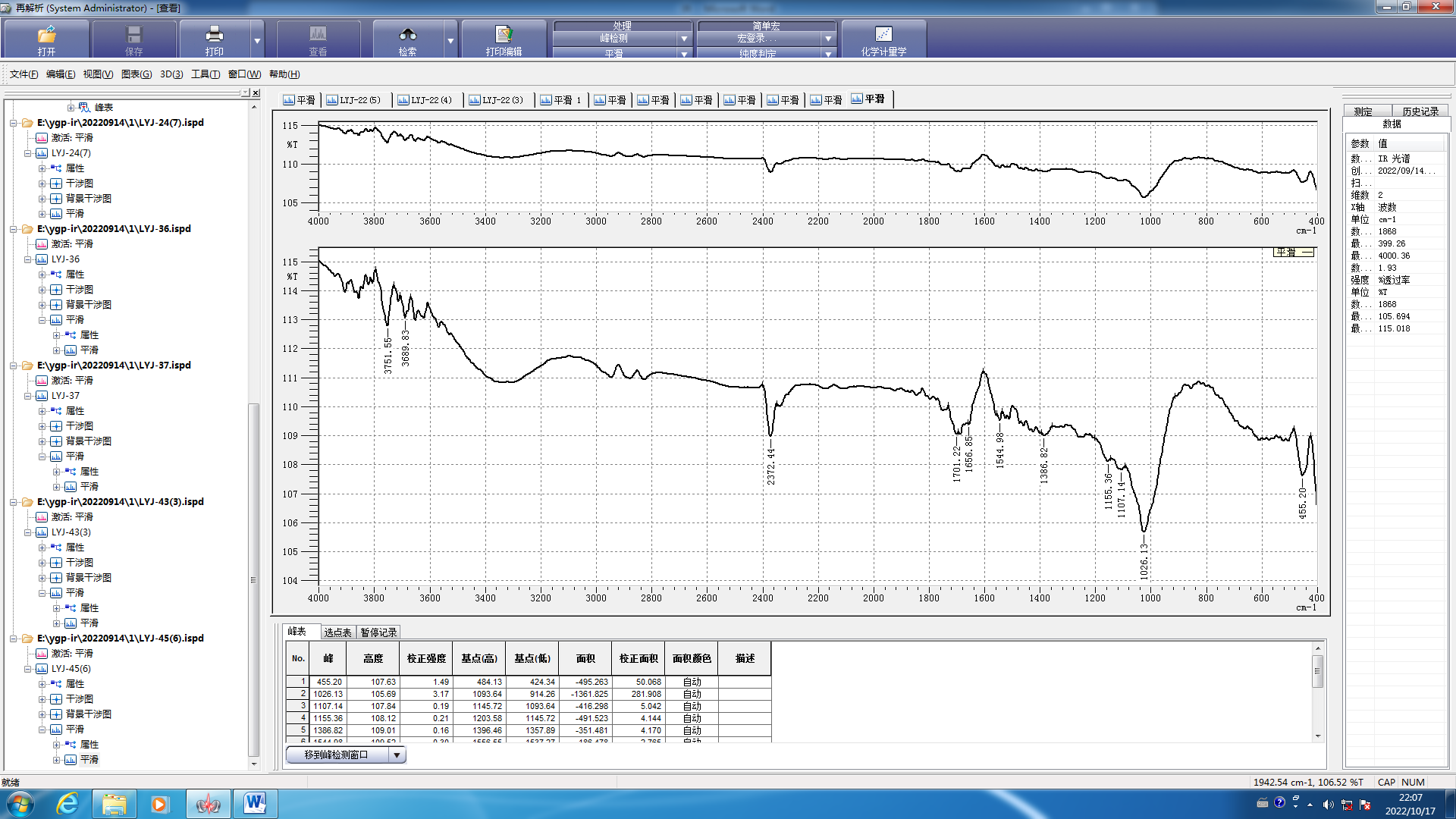


# Figure S8. ^1^H NMR spectrum of compound 2 (400 MHz, CDCl_3_).


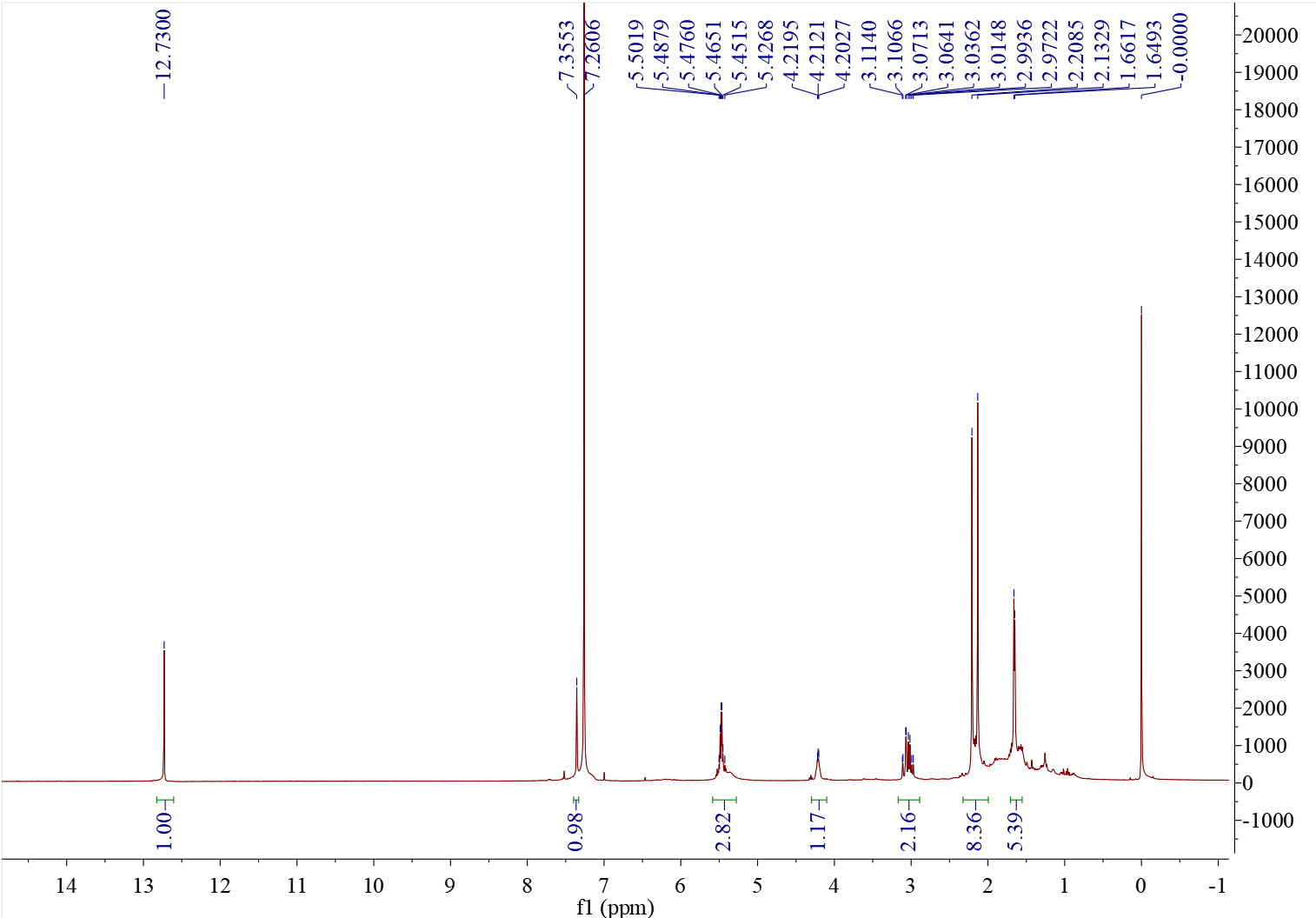


# Figure S9. ^13^C NMR spectrum of compound 2 (100 MHz, CDCl_3_)


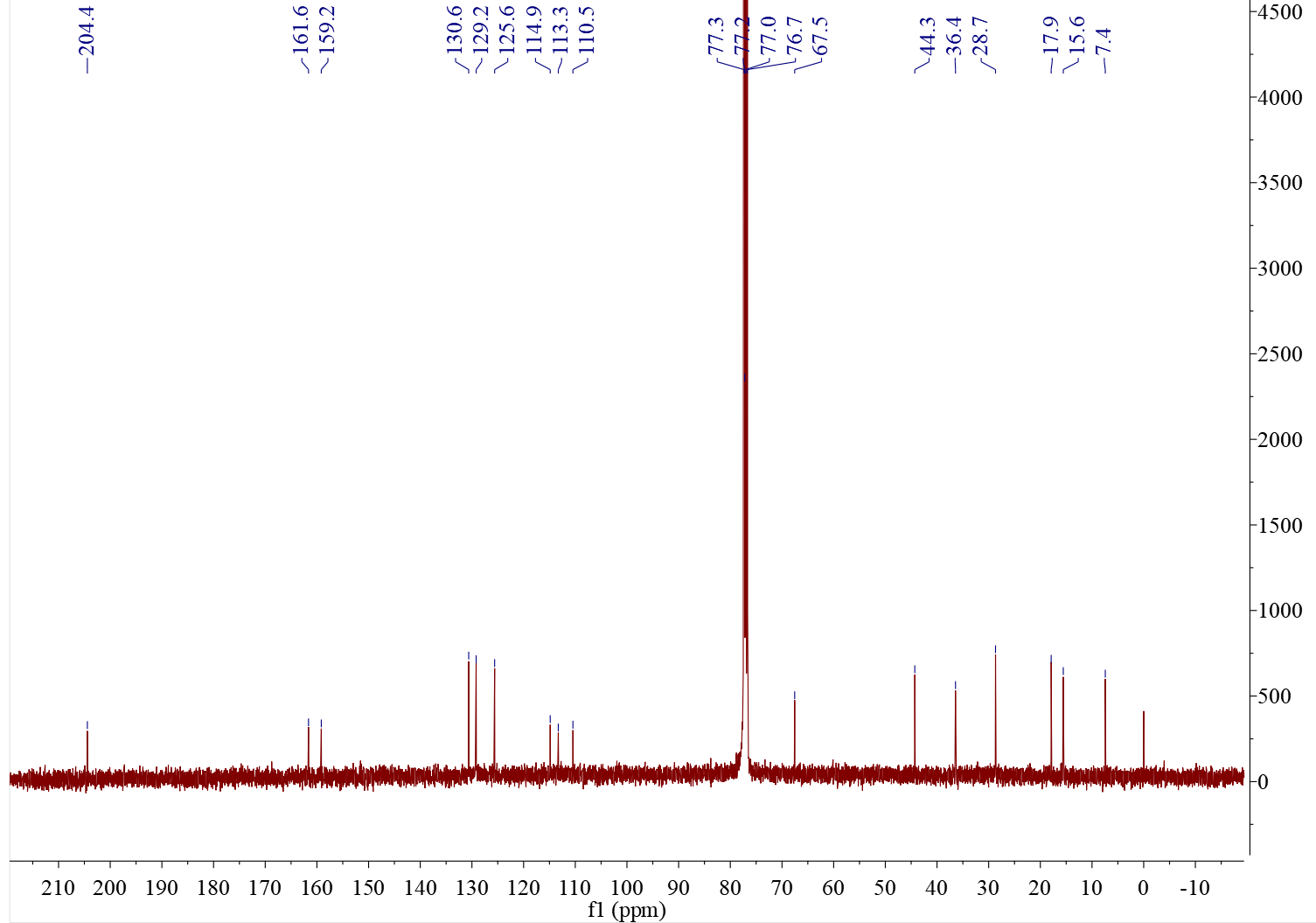


# Figure S10. HSQC spectrum of compound 2 (CDCl_3_)


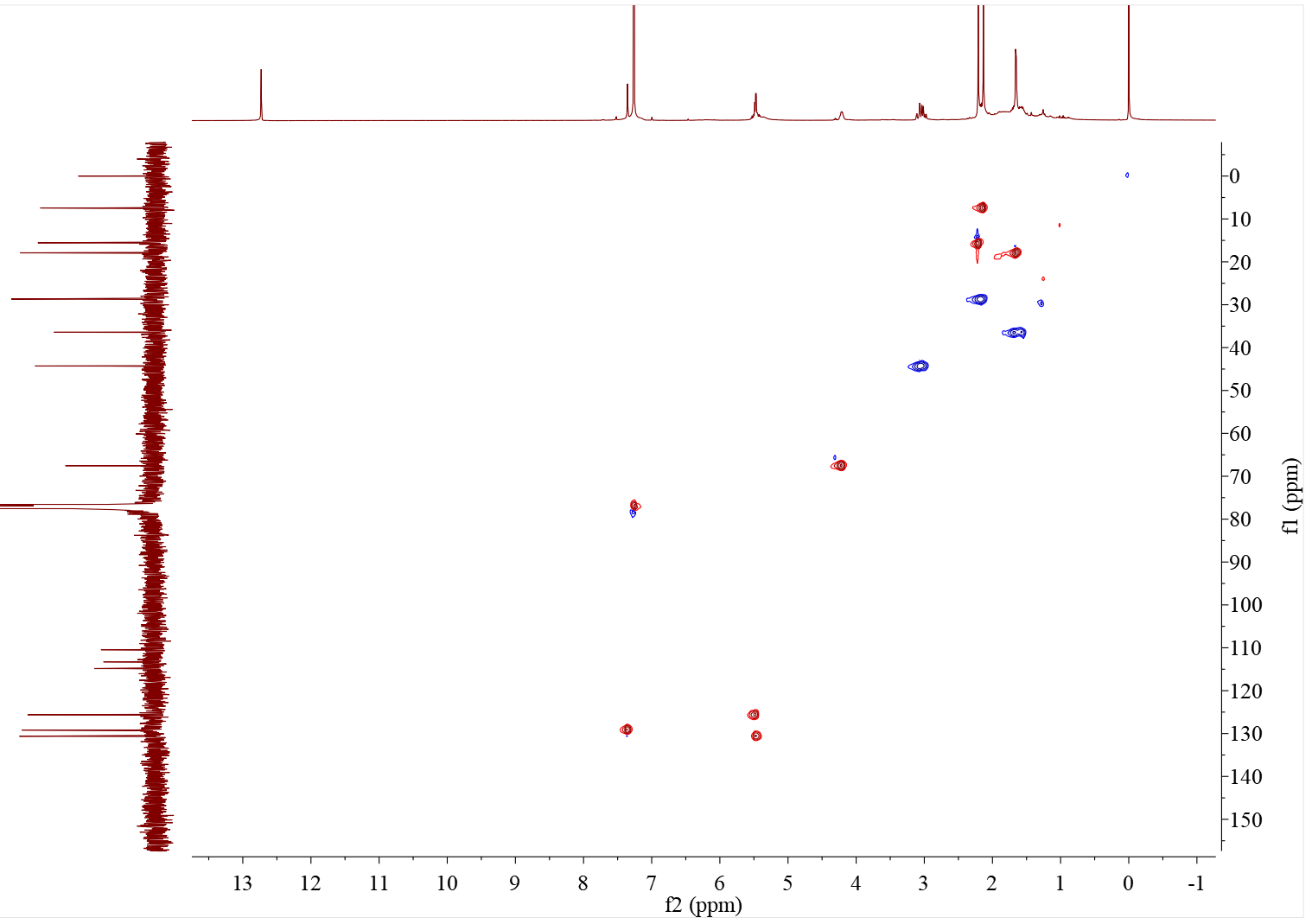


# Figure S11. HMBC spectrum of compound 2 (CDCl_3_)


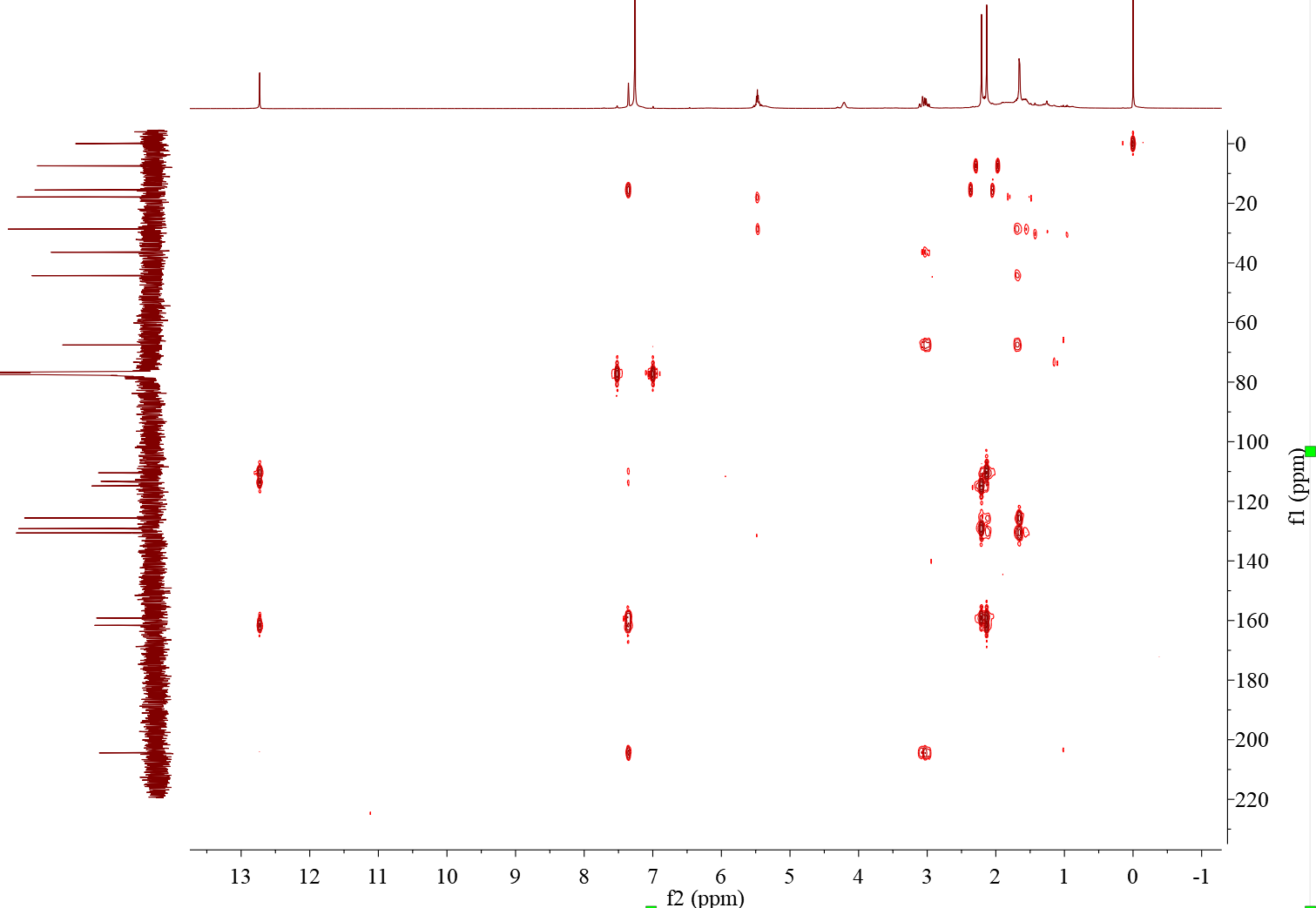


# Figure S12. ^1^H-^1^H COSY spectrum of compound 2 (CDCl_3_)


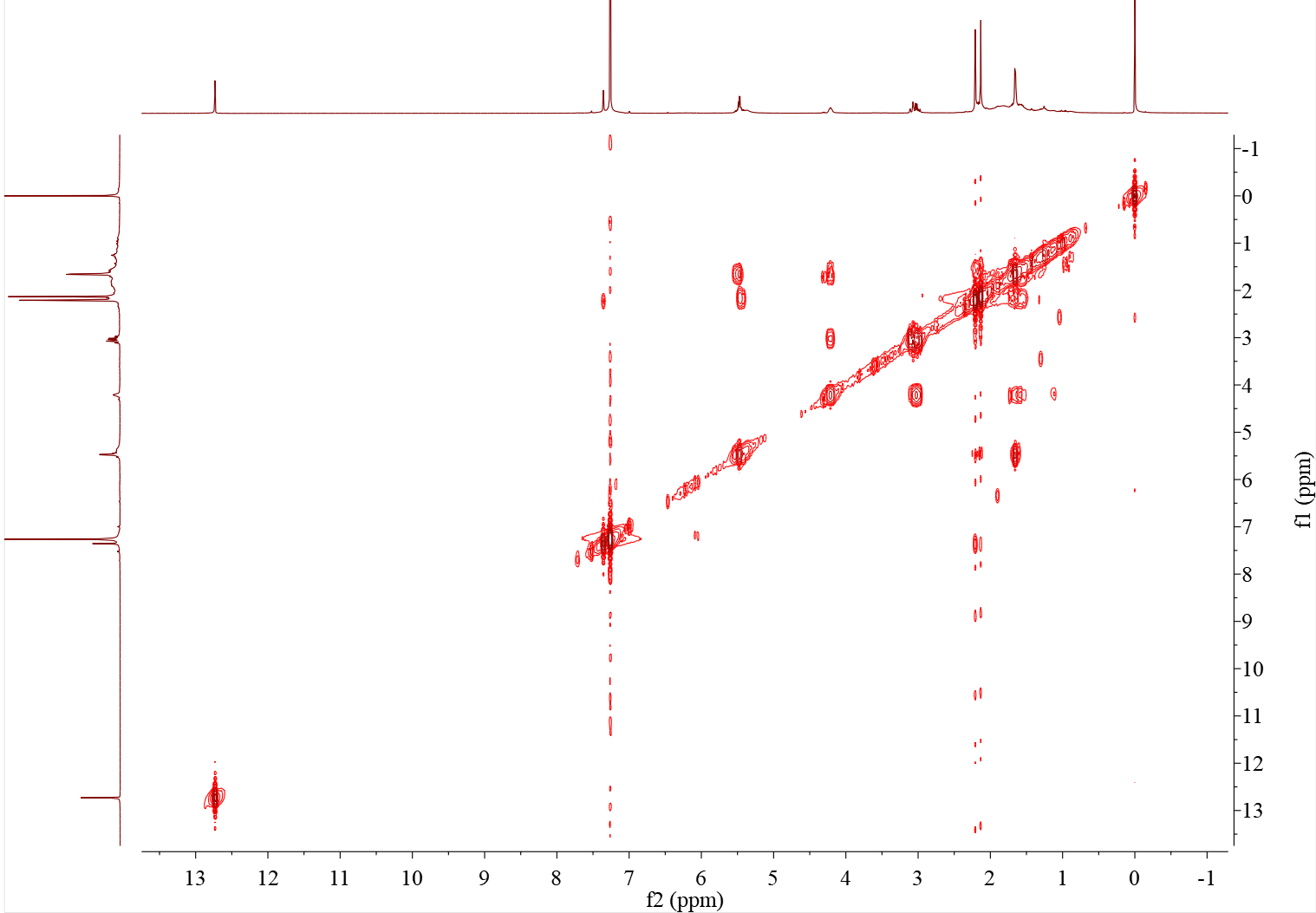


# Figure S13. HRESIMS spectrum of compound 2


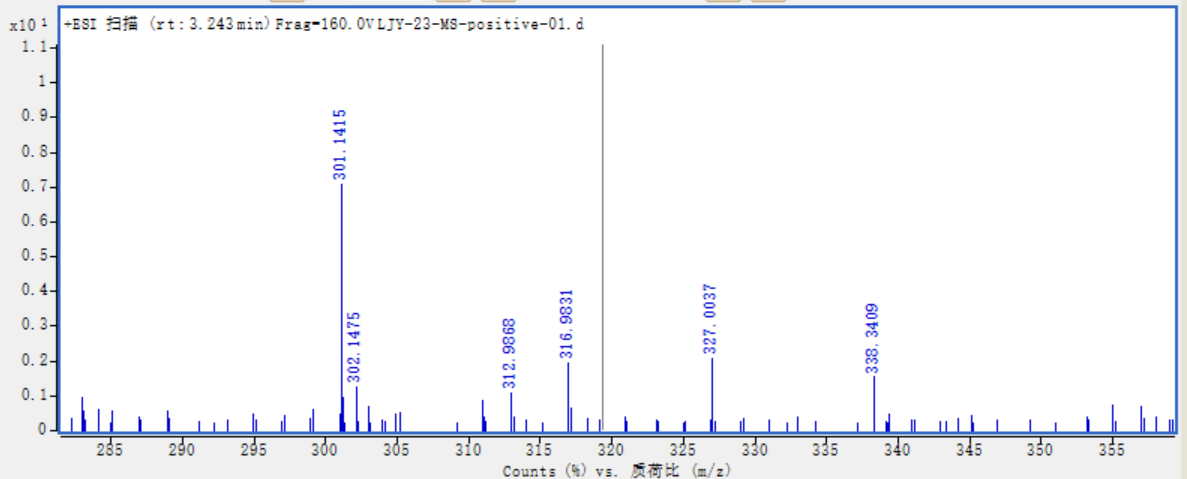


## Figure S14. UV spectrum of compound 2


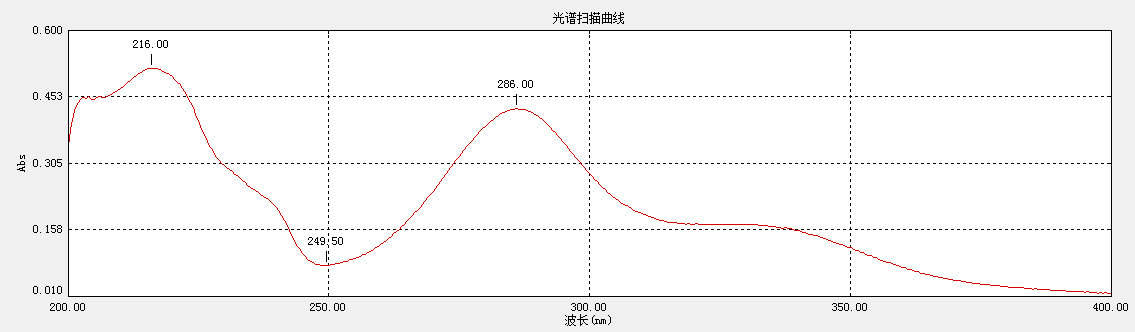


# Figure S15. IR spectrum of compound 2


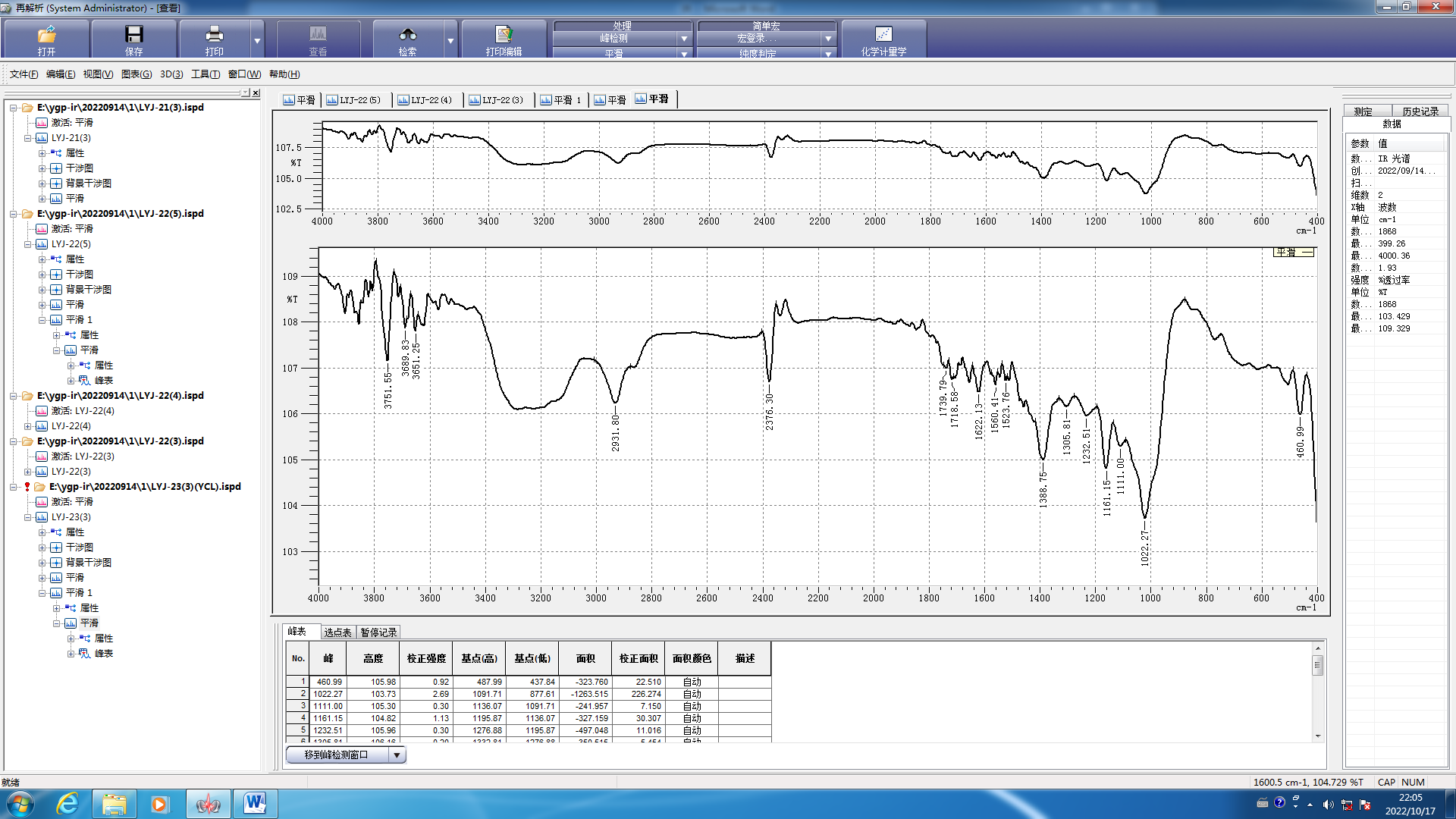


# Figure S16. Experimental and computational ECD spectra of compound 2


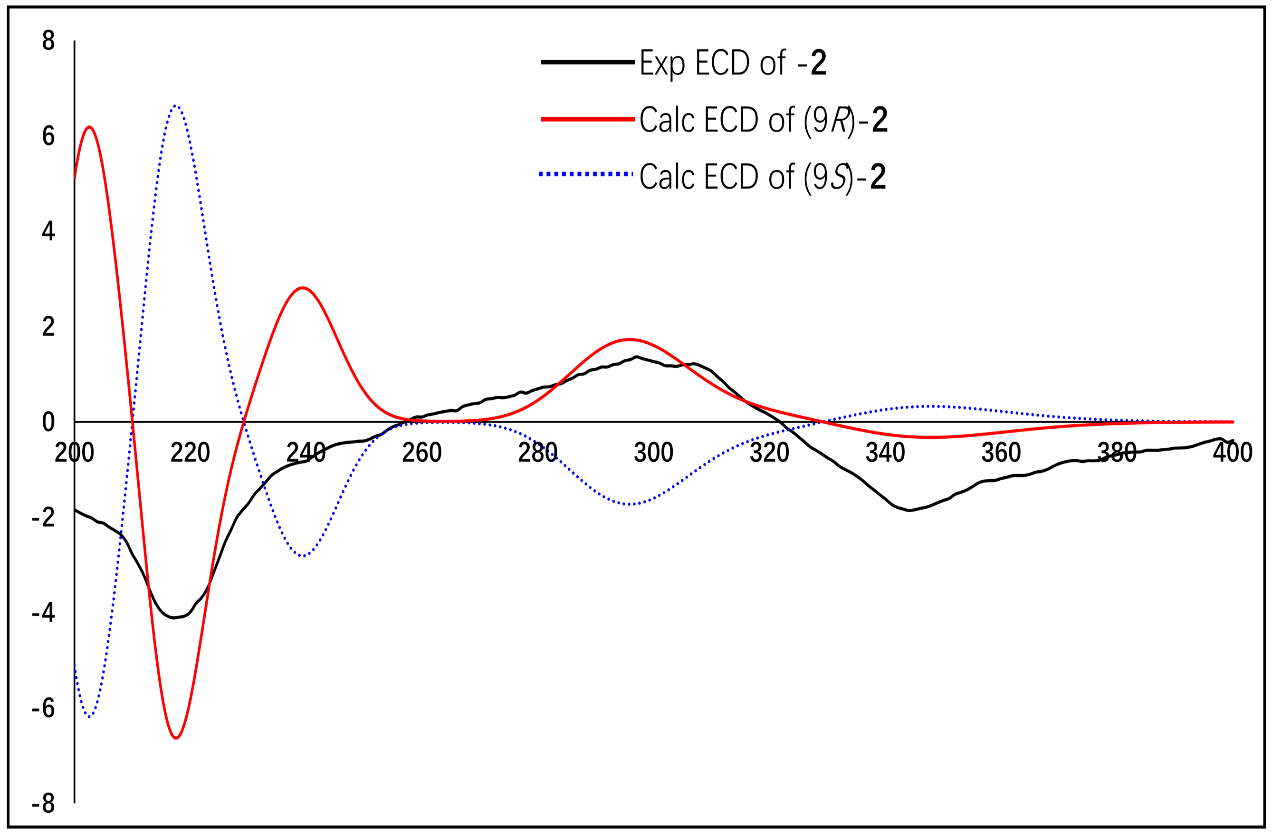


# Figure S17. ^1^H NMR spectrum of compound 3 (400 MHz, CDCl_3_).


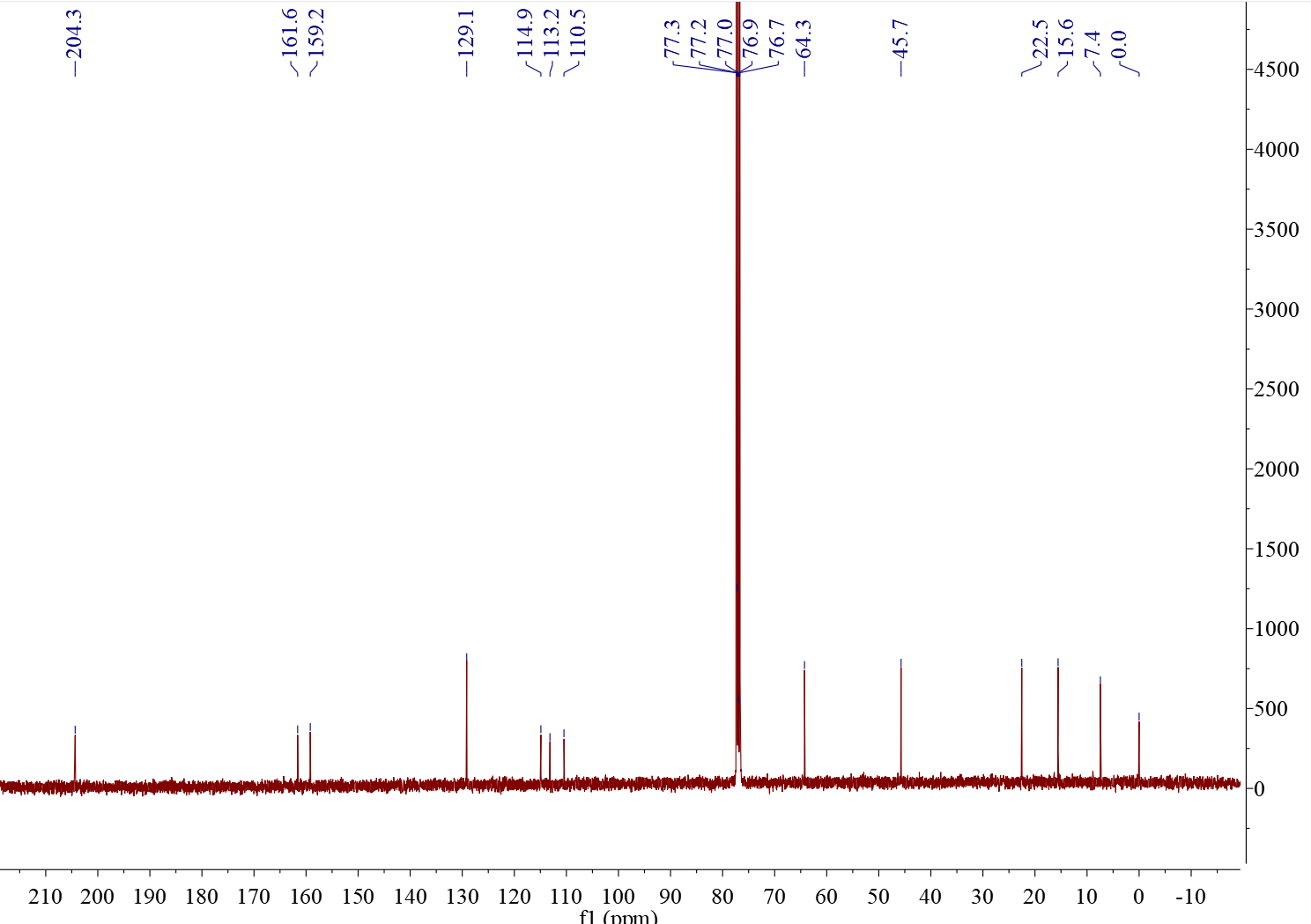


# Figure S18. ^13^C NMR spectrum of compound 3 (100 MHz, CDCl_3_)


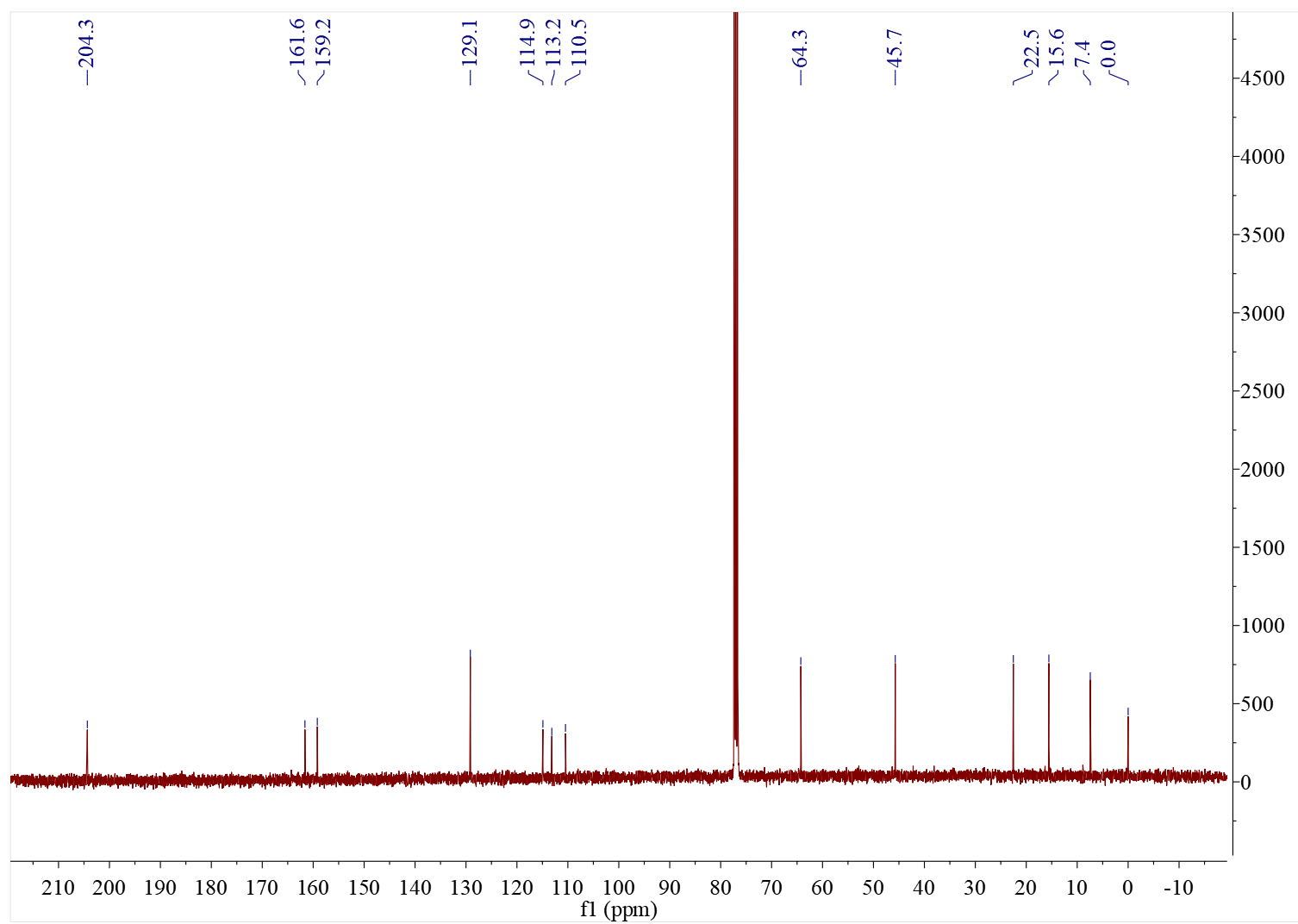


# Figure S19. HSQC spectrum of compound 3 (CDCl_3_)


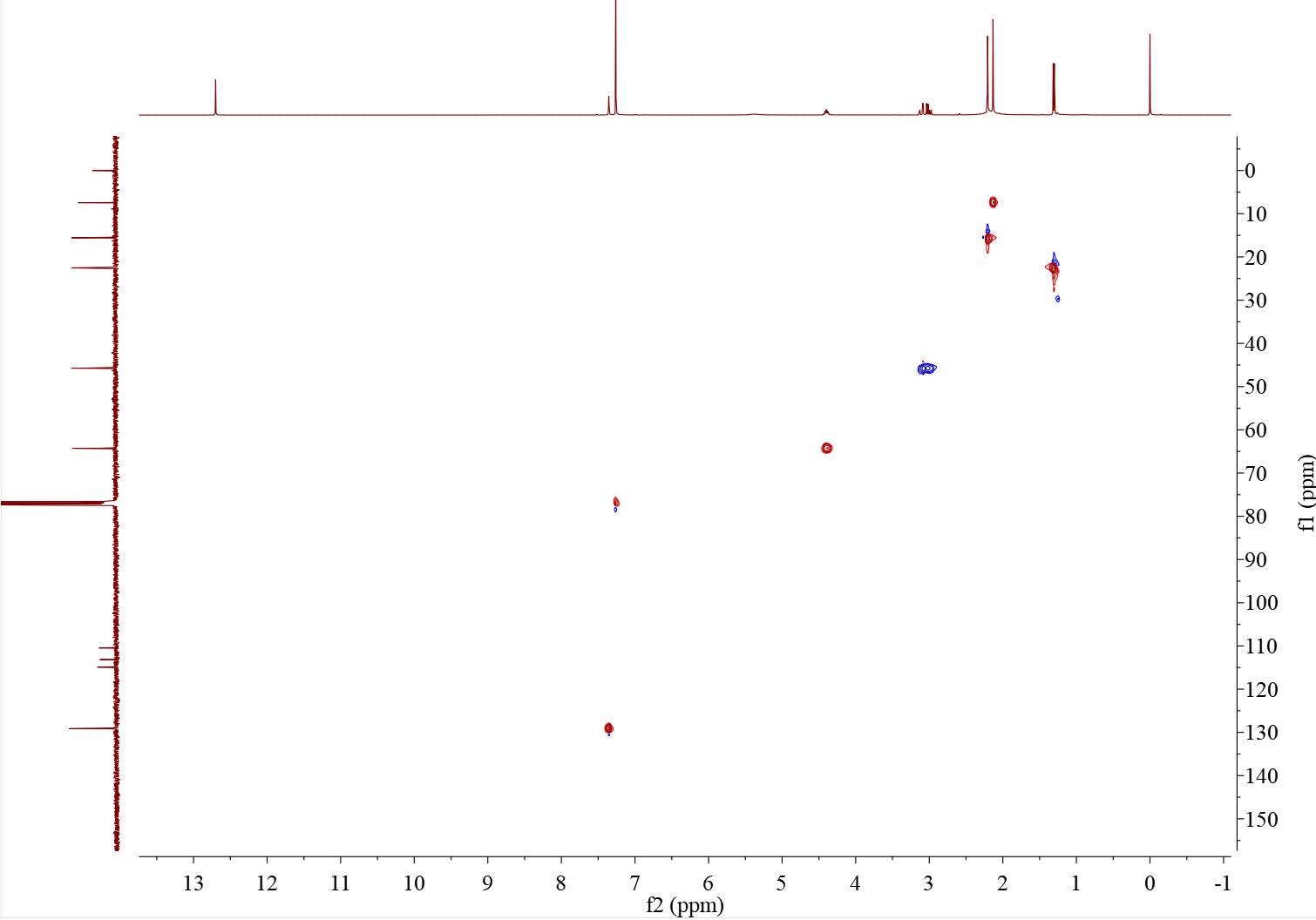


# Figure S20. HMBC spectrum of compound 3 (CDCl_3_)


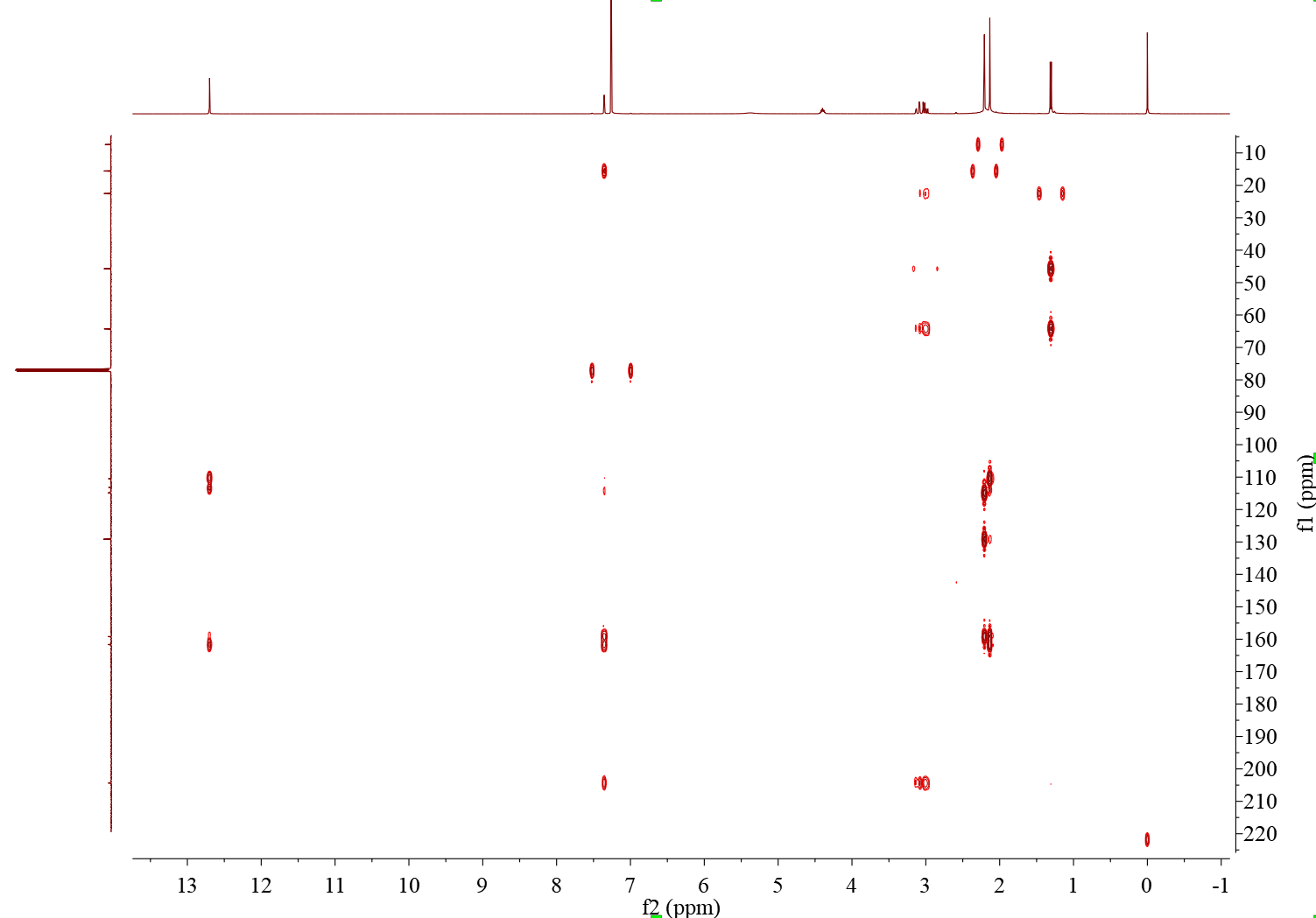


# Figure S21. HRESIMS spectrum of compound 3


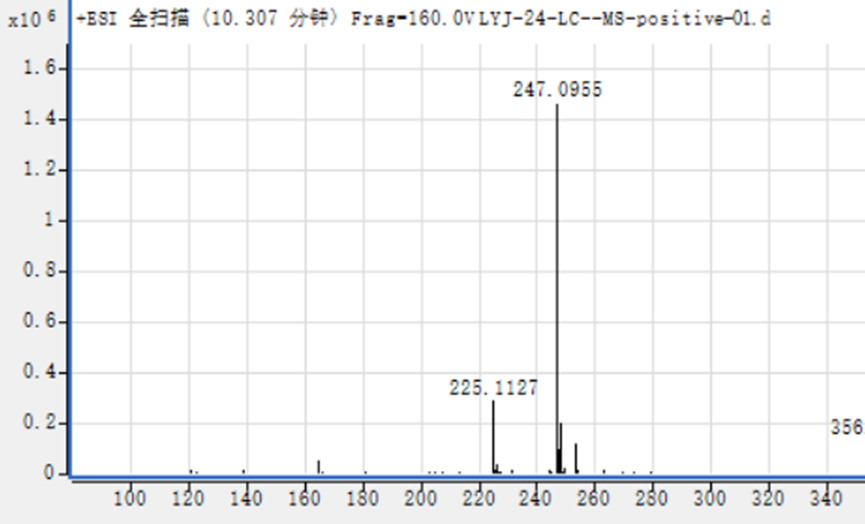


# Figure S22. UV spectrum of compound 3


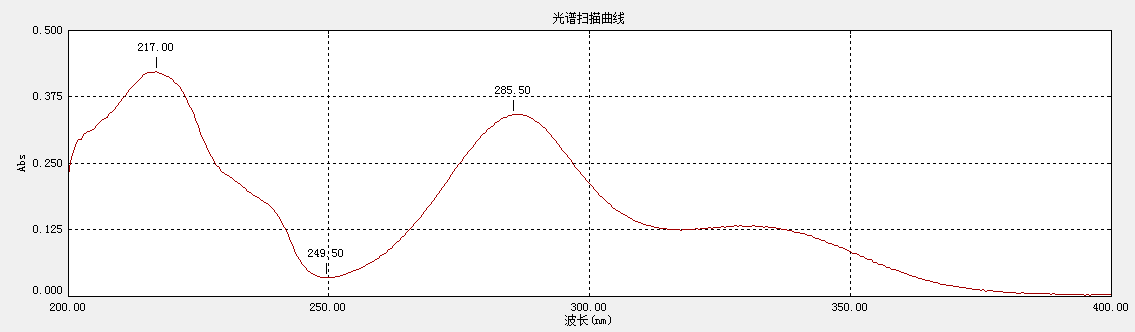


# Figure S23. IR spectrum of compound 3


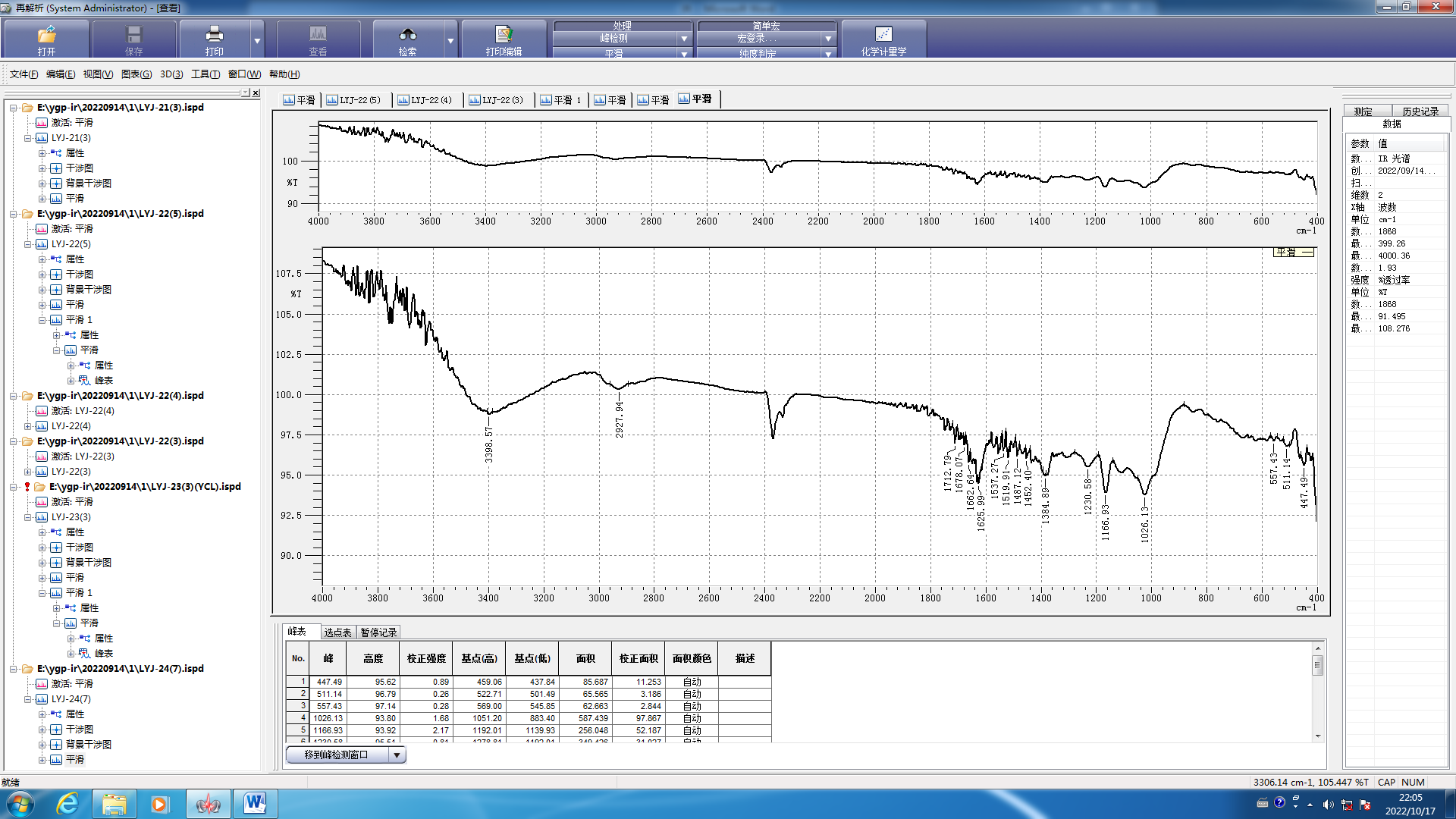


# Figure S24. Experimental and computational ECD spectra of compound 3


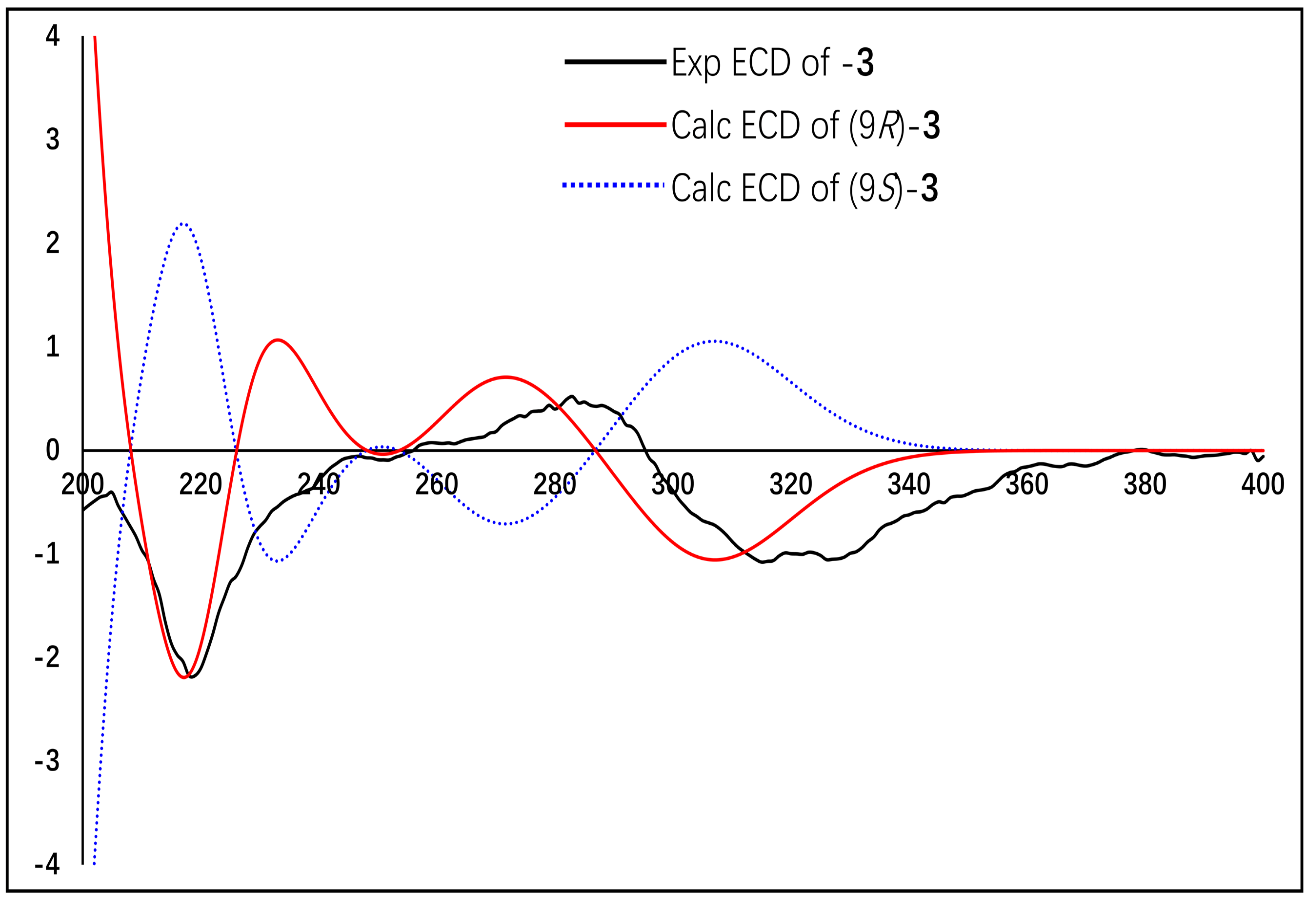


# Figure S25. Generated structures from ACD-Labs.


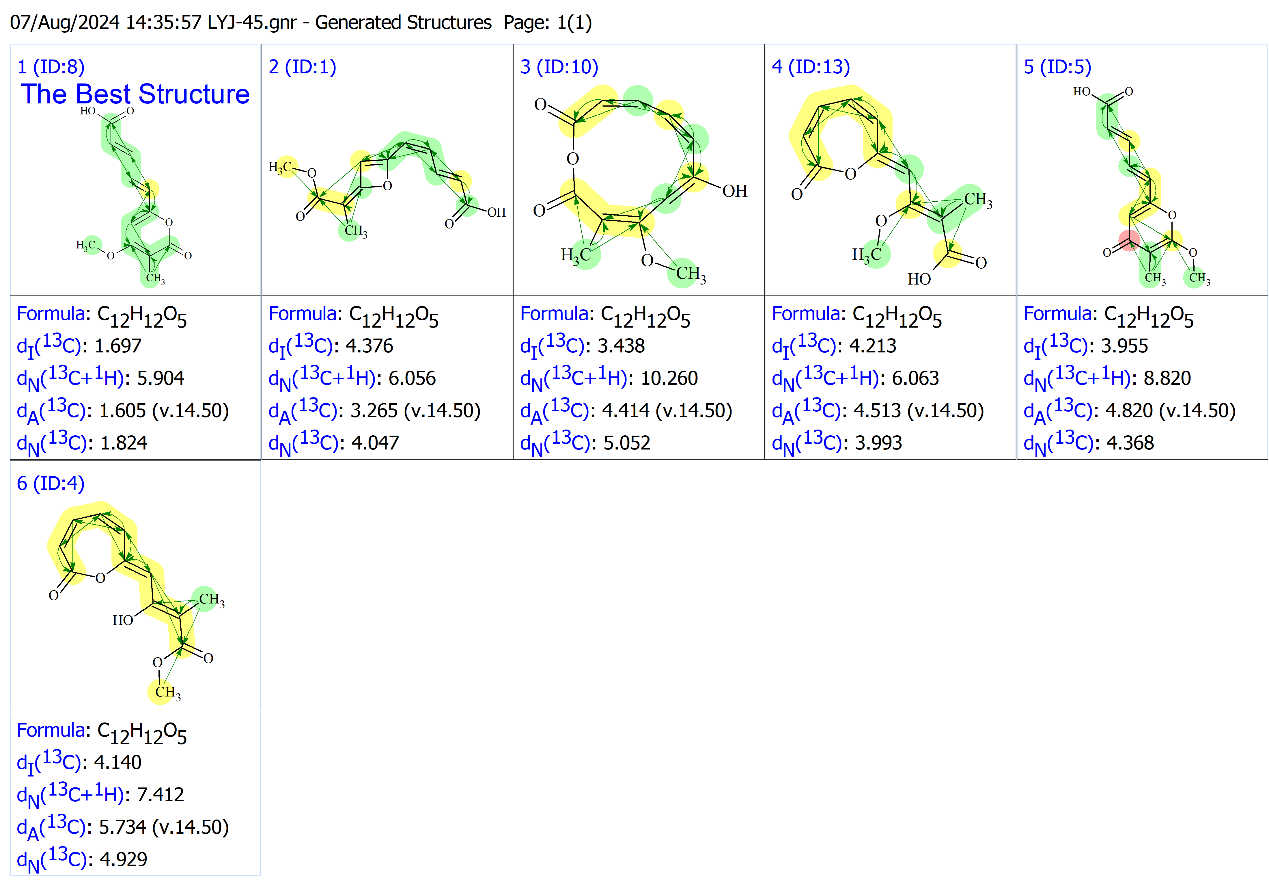


# Figure S26 HPLC chromatogram of compound 1.


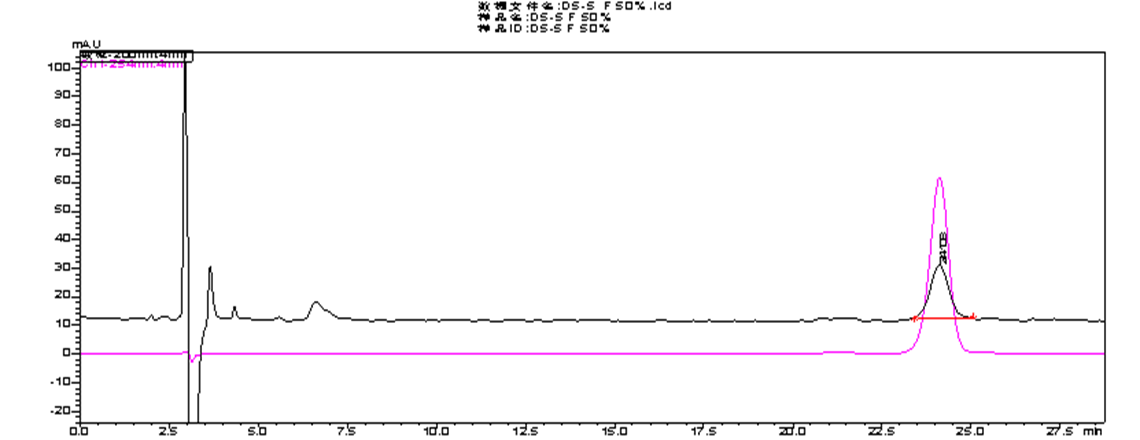


# Figure S27 HPLC chromatogram of compound 2.


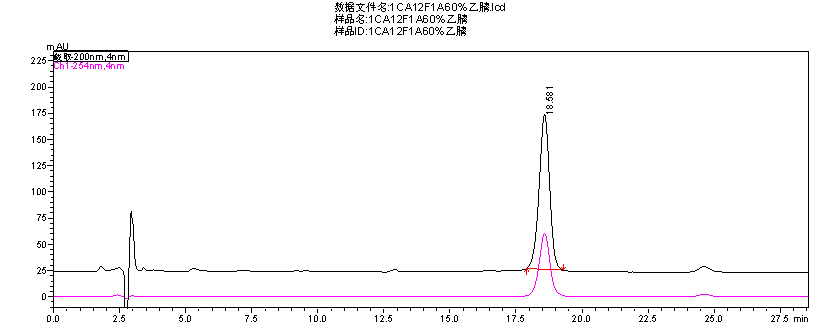


# Figure S28 HPLC chromatogram of compound 3.


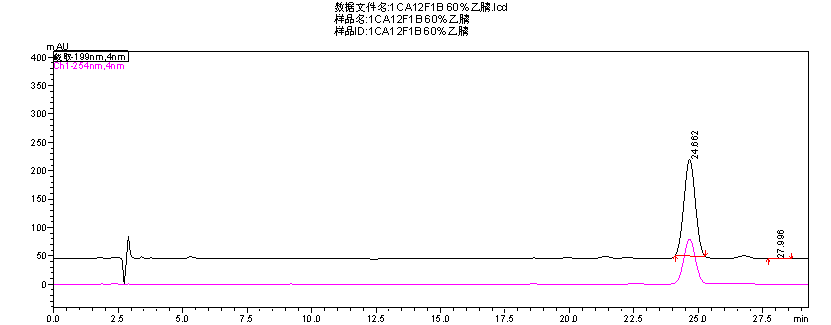


# Figure S29. Colony morphology of *Trichoderma citrinoviride* on PDA after 5 days of growth


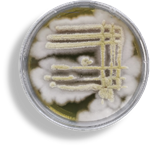


# Figure S30. Microscopic image of *Trichoderma citrinoviride* colony morphology on PDA After 3 days (magnified 40x).


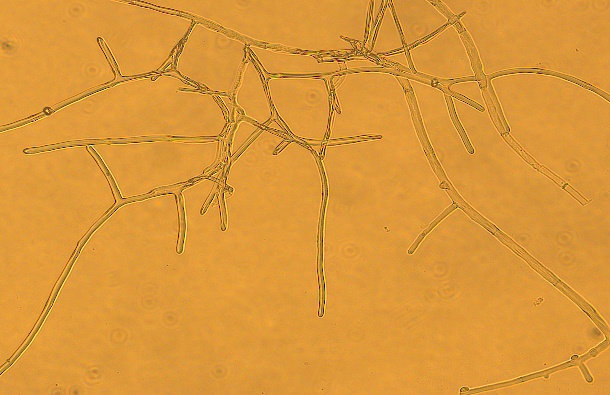

Supplement: Supplementary file 1 [file Data_Sheet_1.docx]
